# A macrophage-specific lncRNA regulates apoptosis and atherosclerosis by tethering HuR in the nucleus

**DOI:** 10.1038/s41467-020-19664-2

**Published:** 2020-12-01

**Authors:** Viorel Simion, Haoyang Zhou, Stefan Haemmig, Jacob B. Pierce, Shanelle Mendes, Yevgenia Tesmenitsky, Daniel Pérez-Cremades, James F. Lee, Alex F. Chen, Nicoletta Ronda, Bianca Papotti, Jarrod A. Marto, Mark W. Feinberg

**Affiliations:** 1 Department of Medicine, Cardiovascular Division, Brigham and Women’s Hospital, Harvard Medical School, Boston, MA USA; 2 Department of Cardiology, The Third Xiangya Hospital of Central South University, Changsha, Hunan China; 3 Feinberg School of Medicine, Northwestern University, Chicago, IL USA; 4 The Blais Proteomics Center, Dana-Farber Cancer Institute, Boston, MA USA; 5 Department of Food and Drug, University of Parma, Parma, Italy; 6 Departments of Cancer Biology and Oncologic Pathology, Dana-Farber Cancer Institute, Boston, MA USA; 7 Department of Pathology, Brigham and Women’s Hospital, Harvard Medical School, Boston, MA USA

**Keywords:** Cell death, Mechanisms of disease, Cardiology, Cardiovascular biology

## Abstract

Long non-coding RNAs (lncRNAs) are emerging regulators of pathophysiological processes including atherosclerosis. Using RNA-seq profiling of the intima of lesions, here we identify a macrophage-specific lncRNA *MAARS* (Macrophage-Associated Atherosclerosis lncRNA Sequence). Aortic intima expression of *MAARS* increases by 270-fold with atherosclerotic progression and decreases with regression by 60%. *MAARS* knockdown reduces atherosclerotic lesion formation by 52% in LDLR^−/−^ mice, largely independent of effects on lipid profile and inflammation, but rather by decreasing macrophage apoptosis and increasing efferocytosis in the vessel wall. *MAARS* interacts with HuR/ELAVL1, an RNA-binding protein and important regulator of apoptosis. Overexpression and knockdown studies verified *MAARS* as a critical regulator of macrophage apoptosis and efferocytosis in vitro, in an HuR-dependent manner. Mechanistically, *MAARS* knockdown alters HuR cytosolic shuttling, regulating HuR targets such as p53, p27, Caspase-9, and BCL2. These findings establish a mechanism by which a macrophage-specific lncRNA interacting with HuR regulates apoptosis, with implications for a broad range of vascular disease states.

## Abstract (web summary)

Long non coding RNA molecules have been implicated in the immunopathology of a range of inflammatory pathologies. Here the authors show lncRNA MAARS regulates apoptosis via interaction with HuR in the context of atherosclerosis.

## Introduction

Atherosclerosis is one of the leading causes of death and disability worldwide. It is a complex disease characterized by lipid accumulation within the arterial wall, inflammation, and apoptosis. Innate immune cells, in particular macrophages, play a pivotal role in atherosclerosis initiation and progression^1,2^. A growing number of studies demonstrate that advanced plaques contain a higher proportion of apoptotic cells than early plaques^3,4^. Accrual of modified LDL can induce macrophage or vascular smooth muscle cell apoptosis, which in earlier plaques are cleared via a process termed efferocytosis^3^. However, as macrophages are triggered into apoptosis, efferocytosis is rendered defective, an effect leading to plaque necrosis and adverse remodeling of the plaque architecture predisposing to vulnerable plaques^5,6^. As such, macrophage apoptosis is a critical contributor to atherosclerosis and plaque necrosis. However, significant gaps remain in the molecular underpinnings that regulate macrophage apoptosis in advanced atherosclerotic plaques.

The recent recognition that <2% of the human genome encodes proteins has opened further opportunities to better understand regulatory pathways in vascular health and disease^7,8^. The majority of biologically active RNAs that cannot be translated into proteins are long non-coding RNAs (lncRNAs) measuring more than 200 nucleotides in length and display mRNA-like characteristics such as being 5′-capped, spliced, and polyadenylated. There is increasing recognition that lncRNAs serve as potent regulators of key cellular processes mediated by their ability to interact with RNA, DNA, proteins, or RNA-binding proteins^9,10^. While there are more lncRNA transcripts than protein-coding genes, their expression, function, and mechanistic roles are poorly defined in atherosclerosis or relevant cell types found in the progression of early or late plaques. Accumulating studies demonstrate that lncRNAs are often enriched in a tissue- or cell-specific manner and regulate significant phenotypic effects^11^, while their subcellular localization pattern can provide additional insights into their mechanistic roles^10^. Discovery of lncRNAs specifically expressed in the intima of atherosclerotic lesions during the progression and regression phases may provide further insights for their roles in atherosclerosis, and potentially decipher mechanisms for apoptosis of advanced lesions.

The RNA-binding protein, HuR (also known as ELAV1) typically binds to AU-rich elements (ARE) to mediate transcript stability, typically of mRNA translation^12,13^. HuR regulates genes involved in key cellular processes such as apoptosis, hypoxia, tumorigenesis, among others^14–16^. HuR function is often linked to its cellular translocation from the nucleus to the cytoplasm^16^. However, a functional role of a specific lncRNA in HuR cytoplasmic shuttling has not been described.

In this study, we have identified and characterized the lncRNA Macrophage-Associated Atherosclerosis lncRNA Sequence (*MAARS*) as an essential regulator of macrophage apoptosis, efferocytosis, and plaque necrosis in atherosclerosis of LDLR^−/−^ mice by direct interaction with the RNA-binding protein HuR, a key mediator of cellular apoptosis. These findings may provide insights into the pathophysiology of a broad range of chronic disease states associated with maladaptive macrophage apoptosis.

## Results

### Discovery of *MAARS* from the intima of atherosclerotic lesions

**Fig. 1:**
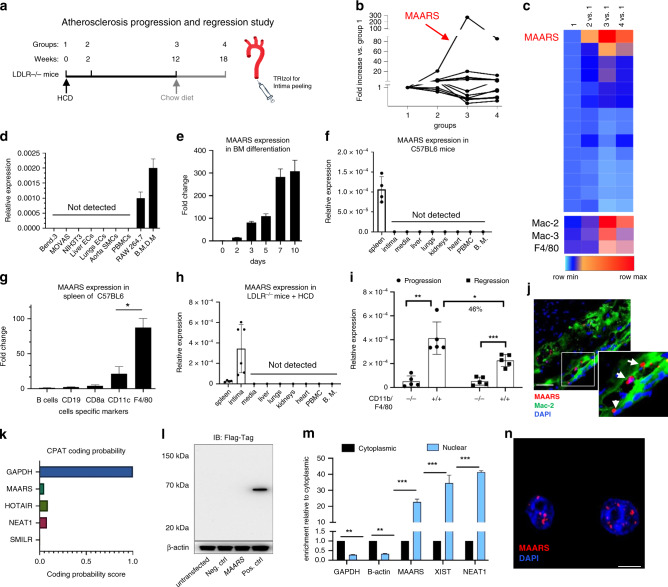
Identification of the lncRNA *MAARS* in lesional intima. **a** RNA derived from aortic intima of LDLR^−/−^ mice (*n* = 3; each sample represents RNA pooled from two mice) that were placed on an HCD for 0 weeks (group 1), 2 weeks (group 2), 12 weeks (group 3), and 18 weeks after 6 weeks of resumption of a normal chow diet (group 4). **b** RNA-Seq results for 11 lncRNA hits obtained by DESeq2/NOR analysis and expressed as fold change compared to group 1 (*n* = 3). **c** Heatmap for 11 lncRNAs that were dynamically regulated with progression and regression of atherosclerosis and expression of macrophage markers Mac2, Mac3, and F4/80, as compared to group 1 (*n* = 3). **d** RT-qPCR expression analysis for *MAARS* in different cell types (*n* = 3). **e** *MAARS* expression kinetics in macrophages differentiated from bone marrow (*n* = 3). **f** *MAARS* expression in body organs and PBMCs of 24 weeks old C57BL/6 mice (*n* = 4, left panel). **g** *MAARS* expression in C57BL6 spleen fractions isolated with magnetic beads specific for different cell types (*n* = 3): F4/80 (macrophages), CD8a (T cells), CD11c (dendritic cells), CD19 (B cells), and negative selection for B cells. **h** *MAARS* expression in body organs and PBMCs of 24 weeks old LDLR^−/−^ mice fed with high cholesterol diet (HCD) for 12 weeks (*n* = 5). **i** *MAARS* expression in 50,000 cells FACS sorted for F4/80- and CD11b-negative or double-positive cells isolated from aorta of ApoE^−/−^ mice fed HCD for 12 weeks (progression) and 18 weeks after 6 weeks of resumption to a normal chow diet (regression) (*n* = 5). **j** RNA*-*in situ hybridization for *MAARS*-probe (red), Mac-2 (green), and DAPI (blue) staining in the aortic lesions of LDLR^−/−^ fed HCD for 12 weeks. **k** Coding potential assessing tool (CPAT) predicts very low coding potential for *MAARS* lncRNA. **l** To test the coding potential, *MAARS* sequence was cloned upstream of 3xFlag-Tag cassette, transfected in 293 T cells, and immunoblotted for Flag antibody. Positive control was provided with the kit (*n* = 3 experiments). **m** RT-qPCR analysis for RNA derived from BMDMs separated into cytoplasmic and nuclear fractions and normalized to the cytoplasmic fraction (*n* = 3). **n** RNA*-*in situ hybridization for negative control- and *MAARS*-probes on PFA-fixed BMDMs. For all panels, values are mean ± SD; *p < 0.05; **p < 0.01; ***p < 0.001.

To discover the presence of lncRNAs in the aortic intima during the progression and regression phases of atherosclerosis, RNA was derived from the aortic intima of LDLR^−/−^ mice after a progression phase of 0, 2, and 12 weeks on high cholesterol diet (HCD) (groups 1–3) and from a regression phase at 18 weeks after 6 weeks of resumption to a normal chow diet (group 4) (Fig. 1a). The aortic intima was used for RNA-Seq profiling to capture differentially expressed lncRNAs (log2-fold change (1.5); FDR < 0.05) and analyzed using DeSeq2 and NOR algorithms as previously described^17^. Both algorithms identified 14 lncRNAs that were commonly dysregulated (Fig. 1b, c). The top differentially regulated transcript was the lncRNA Gm14461 with a 270-fold increase in expression in the aortic intima after 12 weeks of HCD during the progression phase (group 3), which decreased in expression by 60% during lesion regression (group 4), as verified by RT-qPCR (Fig. 1b and Supplementary Fig. 1a). The presence of macrophages and expression of this lncRNA in the aortic intima was further verified by RT-qPCR (Supplementary Fig. 1b, c).

### *MAARS* characterization as a macrophage-specific lncRNA

Gm14461 expression is highly expressed in bone marrow-differentiated macrophages (BMDMs) and the macrophage cell line RAW264.7 (Fig. 1d). In contrast, the Gm14461 transcript was undetectable in all the other cells types tested, including endothelial cells (primary isolated endothelial cells from liver and lung, and the endothelial cell line Bend.3), vascular smooth muscle cells (primary mouse aortic smooth muscle cells and the MOVAS cell line), NIH3T3 fibroblasts, or freshly isolated bone marrow or peripheral blood mononuclear cells (PBMCs) (Fig. 1d). While Gm14461 transcript is undetectable in mouse bone marrow, its expression increases exponentially up to 283-fold after 7 days of differentiation in the presence of macrophage colony stimulating factor (MCSF) (Fig. 1e). Similar trends were observed in macrophages differentiated from PBMCs and spleen cells (Supplementary Fig. 1d). For this reason, we named this lncRNA *MAARS*. *MAARS* was detected only in the spleens of C57BL/6 mice, where a high number of macrophages are located, whereas it was undetectable in all other organs (Fig. 1f). Moreover, we have isolated spleens and sorted different cell populations using magnetic beads labeled with specific antibodies such as F4/80 (macrophage marker), CD11c (dendritic cells and macrophages), CD8a (T cells), CD19 (B cells), and negative sorting of B cells, and found that *MAARS* was highly enriched in the F4/80-sorted fraction (Fig. 1g). In contrast, the highest expression of *MAARS* was detected in the intima of LDLR^−/−^ mice fed HCD for 12 weeks (12-fold higher than the spleen, Fig. 1h), in accordance with the abundance found in our initial RNA-seq screening data (Fig. 1b, h). To further assess the regulation of *MAARS* expression specifically in the macrophages with different phases of atherosclerosis (Fig. 1b, c), FACS-sorted F4/80^+^/CD11b^+^ macrophages were isolated during atherosclerosis progression and regression. *MAARS* expression was eightfold increased in the F4/80^+^/CD11b^+^ double-positive population of macrophages compared to the negative-stained cells in the progression group, while in the atherosclerosis regression group *MAARS* expression decreased by 46% in F4/80^+^/CD11b^+^ macrophages (Fig. 1i). To obtain additional verification for its macrophage specificity, *MAARS* (nuclear staining) was visualized by RNA-in situ hybridization (ISH) (red) in close proximity to the cytoplasmic macrophage marker Mac-2 (antibody-stained in green) in the aortic sinus (Fig. 1j and Supplementary Fig. 1e).

In 5′RACE PCR studies, we confirmed the six exons currently annotated for *MAARS* in the NCBI and Ensemble databases, in accordance with RNA-seq data (Supplementary Fig. 1f). Intriguingly, *MAARS* is annotated as a lncRNA in the Ensembl database, while in NCBI it is annotated as an mRNA. Hence, we sought to elucidate this transcript by first checking the coding probability using the in silico coding potential assessment tool (CPAT) and found that *MAARS* has a similar score to other well-described lncRNAs such as HOTAIR, NEAT1, or SMILR (Fig. 1k). Consequently, for in vitro validation of peptide coding potential, *MAARS* sequence was cloned upstream of the p3xFLAG-CMV plasmid, transfected in HEK293 cells, and immunoblotted for FLAG Tag, which showed no detectable peptide or protein (Fig. 1l). In addition, *MAARS* is polyadenylated (Supplementary Fig. 1g) and enriched in the nucleus of the macrophages as observed by cell fractionation (Fig. 1m) and by RNA-ISH in bone marrow-derived macrophages (Fig. 1n and Supplementary Fig. 1e). *MAARS* is highly expressed in BMDMs with 9.84 × 10^8^ copies ng^−1^ of RNA and is expressed 2.4-fold higher than the regularly used housekeeping gene hypoxanthine phosphoribosyltransferase 1 (HPRT) (Supplementary Fig. 1h). Collectively, these results indicate that *MAARS* is a macrophage- and nuclear-specific lncRNA markedly induced with atherosclerotic progression.

### *MAARS silencing* reduces progression of atherosclerosis

**Fig. 2:**
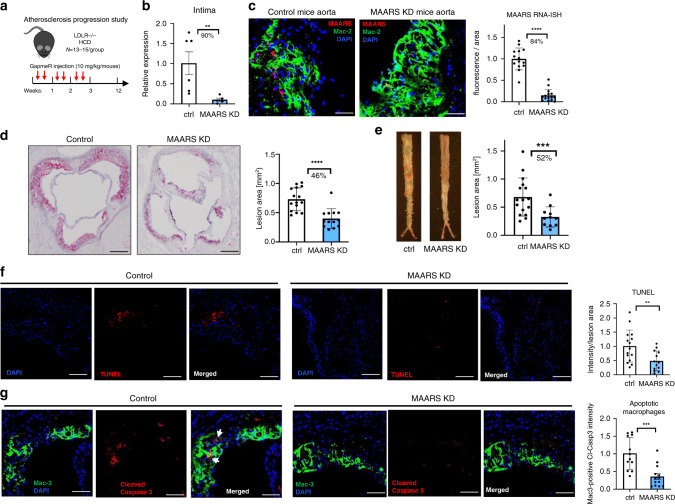
In vivo knockdown of *MAARS* inhibits atherosclerotic lesion formation. **a** LDLR^−/−^ mice were i.v. injected with vehicle control gapmeR (*n* = 15) or *MAARS* gapmeR (*n* = 13) twice per week (7.5 mg kg^−1^ per injection per mouse) and placed on HCD for 12 weeks. **b** Silencing of *MAARS* was assessed by RT-qPCR in RNA derived from the aortic intima (*n* = 6 for control gapmeR group and *n* = 5 for *MAARS* gapmeR group). **c** RNA-ISH representative images and quantification of *MAARS* knockdown in Mac2 co-stained aortic sinus from control and *MAARS* KD groups of mice. Representative images and quantification for Oil-Red O staining in the lesions of aortic sinus (**d**) and in the descending aorta (**e**) of the control (*n* = 15) and *MAARS* KD (*n* = 13) mice. **f** TUNEL (red) staining was detected in the aortic sinus of the control (*n* = 15) and *MAARS* knockdown (KD, *n* = 13) groups of LDLR^−/−^ mice fed HC, counterstained with DAPI (blue), and quantified as total red fluorescent staining per lesion area. **g** The aortic sinus was co-stained with cleaved caspase-3 (red) and Mac-3 antibodies (green) and the Mac3-associated caspase-3 fluorescence was quantified in the aortic sinus of the control (*n* = 15) and *MAARS* KD (*n* = 13) mice. For all panels, values are mean ± SD; *p < 0.05; **p < 0.01; ***p < 0.001; ****p < 0.0001; *****p < 0.00001.

For *MAARS* silencing we designed different LNA-based gapmeRs that showed 70–90% knockdown efficiency in BMDMs and selected gapmeR #1 for further studies (Supplementary Fig. 1i). To explore the role of systemically delivered *MAARS*-gapmeRs in atherosclerosis, LDLR^−/−^ mice were i.v. injected with vehicle control or *MAARS* gapmeR twice per week (10 mg kg^−1^ per injection per mouse) over 12 weeks on HCD (Fig. 2a). After 12 weeks on HCD, gapmeR-mediated silencing of *MAARS* reduced its expression in the aortic intima by 90% (Fig. 2b) and in the spleens by 40% (Supplementary Fig. 4f), while *MAARS* could not be detected in the aortic media and the other organs (Fig. 1h and Supplementary Fig. 1b). Furthermore, the *MAARS*-specific knockdown in the intima was confirmed by RNA-ISH in the aortic sinus lesions, showing an 84% decrease (Fig. 2c). Remarkably, analysis of atherosclerotic lesion formation by Oil-Red O (ORO) staining revealed a 46% decrease in lesion area in the aortic sinus and 52% decrease in the descending thoracoabdominal aorta after knockdown of *MAARS* (Fig. 2d, e). As shown by immunohistochemistry, *MAARS*-deficient lesions did not differ in the accumulation of macrophages (Mac-2 and Mac-3), CD4^+^ or CD8^+^ T cells, MHCII-positive cells, or vascular smooth muscle cells (Supplementary Fig. 2) after normalization to lesion area.

While *MAARS* knockdown was associated with a modest reduction in total cholesterol (22%), LDL (23%), HDL (20%), and triglycerides (18%) (Supplementary Fig. 3a), the lesion areas as quantified by ORO staining were still reduced by 37% in *MAARS* knockdown mice when examined in mice with similar total cholesterol in both groups (Supplementary Fig. 3b). Thus, although ∼9% of the atherosclerotic plaque reduction may be accounted for the serum cholesterol level reduction, this cannot entirely explain the substantial reduction in atherosclerosis lesions. Nevertheless, we explored a possible lipid-related mechanism impacting plaque formation and found that *MAARS* can also affect macrophage cholesterol handling. *MAARS* knockdown in BMDMs increased cholesterol efflux to the main cholesterol acceptors, HDL, ApoA-1, and to normal serum (Supplementary Fig. 3c). These data suggest that the modest contribution to the plaque reduction by MAARS deficiency may be due to cholesterol handling.

Although *MAARS* was undetectable in PBMCs, lesional macrophages are in part differentiated from recruited PBMCs, thereby monocyte polarization was analyzed for Ly6C inflammatory expression by FACS in both PBMCs and the spleen. No differences were observed in the anti-inflammatory Ly6C^low^ or the pro-inflammatory Ly6C^interm^ or Ly6C^high^ fractions in the spleens and PBMCs of the *MAARS*-knockdown group compared to vehicle control (Supplementary Fig. 4a). While antisense oligonucleotides can induce liver toxicity^18^, *MAARS* in vivo knockdown had no significant effect on mouse body weights and the aspartate transaminase (AST) serum levels (Supplementary Fig. 4b, c). Although there were modest increases in the alanine transaminase (ALT) levels, these were in the normal accepted physiological range for C57BL/6 mice, e.g., below 70 IU l^−1^ (Supplementary Fig. 4d)^19,20^. RNA expression of MCP-1, IL-6, and TNF-α pro-inflammatory markers were reduced in aortic lesions, but there were no differences observed in the expression of inflammatory genes IL-1β and COX-2 in the aortic intima (Supplementary Fig. 4e). Furthermore, only MCP-1 was reduced in the spleens of *MAARS* knockdown group, while IL-1β, IL-6, TNF-α, and MIP-1B were not significantly regulated (Supplementary Fig. 4g). Importantly, there were no regulatory effects on the activity of pro-inflammatory signaling pathways NF-Kβ, p38- and ERK1/2-MAPK, Akt, or JNK in BMDMs after *MAARS* gapmeRs transfection (Supplementary Fig. 4h). Taken together, these findings indicate that *MAARS* deficiency markedly reduced atherosclerotic lesion formation with a mild effect on inflammatory markers and lipid metabolism, and with acceptable liver toxicity.

### *MAARS* knockdown decreases macrophage apoptosis

**Fig. 3:**
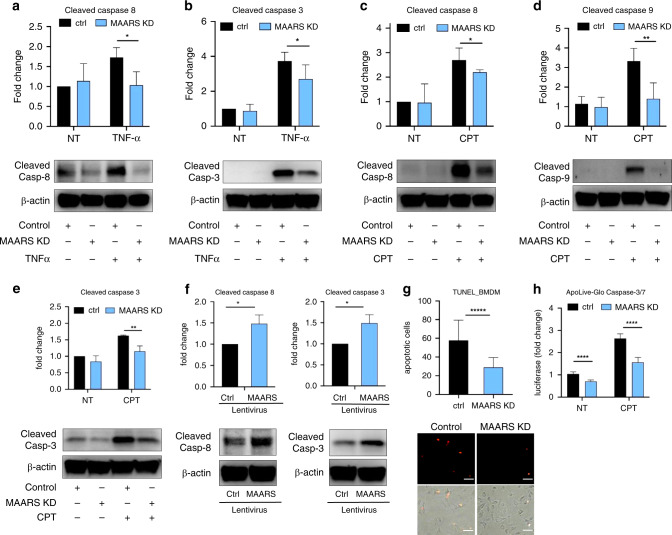
*MAARS* knockdown decreases macrophage apoptosis. Western blot quantification of cleaved caspase-8 (**a**) and cleaved caspase-3 (**b)** lysate from BMDMs transfected with *MAARS*-specific or control gapmeRs, and activated with TNF-alpha (50 ng ml^−^^1^) for 18 h (*n* = 4). Western blot quantification of cleaved caspase-8 (**c**), cleaved caspase-9 (**d**), and cleaved caspase-3 (**e**) from lysates of BMDMs transfected with *MAARS*-specific or control gapmeRs, and activated for 2 h with 20 µM camptothecin (CPT; *n* = 3). **f** Cleaved caspase-3 and -8 expression in BMDMs transduced with lentivirus control or overexpressing *MAARS* (*n* = 3). **g** In vitro TUNEL staining of BMDMs transfected with *MAARS*-specific and control gapmeRs and activated with 20 µM CPT (*n* = 3; 25 images quantified per condition). **h** Quantification of apoLiveGlo cleaved caspase-3/7 enzymatic assay of BMDMs transfected with *MAARS*-specific and control gapmeRs and activated with or without CPT (20 µM) (*n* = 3) For all panels, values are mean ± SD; *p < 0.05; **p < 0.01; ***p < 0.001; ****p < 0.0001; *****p < 0.00001.

To investigate additional mechanisms by which neutralization of *MAARS* reduced atherosclerotic lesion formation, we investigated the possibility that *MAARS* could affect lesional apoptosis. Accumulating studies highlight that apoptosis of lesional macrophages plays a crucial role in atherosclerosis, dependent on the stage of progression^5,21^. Investigation of lesion apoptosis by terminal deoxynucleotidyl transferase dUTP nick-end labeling (TUNEL) assay showed a significant reduction in apoptosis (by 52%) in the aortic sinus of the *MAARS*-knockdown group (Fig. 2f). Moreover, co-staining of Cleaved Caspase-3 and macrophage marker Mac-2 revealed a decrease of apoptotic macrophages (by 55%) in the lesions (Fig. 2g). To further assess whether *MAARS* directly regulates apoptosis in BMDMs, we performed *MAARS* knockdown in the presence and absence of apoptotic stimuli. Indeed, *MAARS* knockdown dramatically reduced the levels of cleaved Caspase-8 and -3 in BMDMs treated with high-dose TNF-α to trigger apoptosis, and of cleaved Caspase-8, -9, and -3 in BMDM stimulated with Camptothecin (CPT), a topoisomerase I inhibitor that activates apoptosis by inducing DNA damage (Fig. 3a–e)^22^. Similar results were obtained when *MAARS* was silenced with a different *MAARS* gapmeR (Supplementary Fig. 5h). In contrast, *MAARS* overexpression in BMDMs using a lentivirus system increased the protein levels of cleaved Caspase-3 and -8 (Fig. 3f). In a complementary approach, *MAARS* silencing decreased macrophage apoptosis by 50% as quantified by the TUNEL assay in CPT-treated BMDMs (Fig. 3g). Finally, we used the ApoLive-Glo Caspase-3/7 enzymatic assay that detects the cleavage of Caspases-3 and -7 and observed decreased levels of luciferase activity in the *MAARS*-knockdown macrophages by 30% under basal conditions and by 40% in CPT-treated cells (Fig. 3h). Collectively, these findings suggest that *MAARS* has potent effects on regulating macrophage apoptosis in vitro and in vivo.

### *MAARS* interacts with HuR (ELAVL1) to regulate apoptosis

**Fig. 4:**
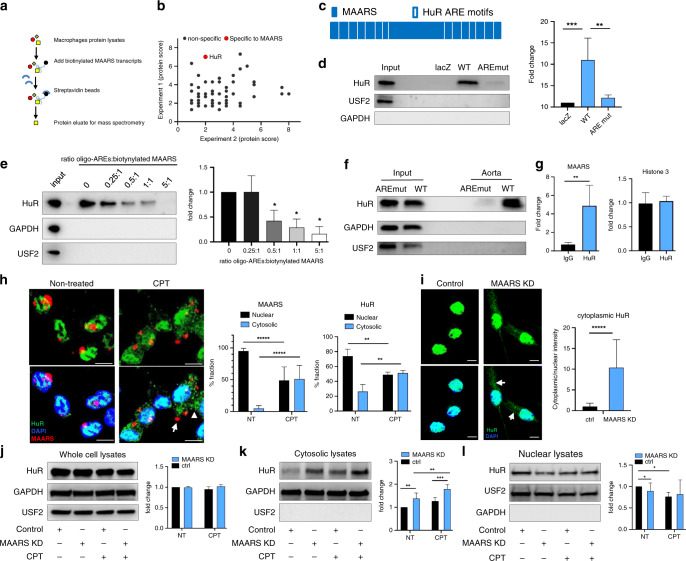
*MAARS* interacts with HuR (ELAVL1). **a** Illustration of lncRNA pulldown using nuclear protein extract from RAW264.7 macrophages for in vitro transcribed biotin-labeled *MAARS* or negative control LacZ followed by streptavidin beads pulldown. Protein eluate was sent for MS. **b** Proteins identified in pulldowns of biotynlated *MAARS* from two independent experiments, two technical replicates. **c** Schematic representation of HuR binding motifs to AU-rich elements (ARE) on *MAARS* transcript. **d** Biotinylated lncRNA pulldown using *MAARS* wild type (WT) or *MAARS* with mutated HuR binding ARE sequences (AREmut) in nuclear lysates of BMDMs (*n* = 5). **e** Competition pulldown using biotinylated *MAARS* and RNA synthetic AREs oligonucleotides at different picomole ratios (*n* = 3). **f** In vivo lncRNA pulldown using whole cell lysates from aorta of ApoE^−/−^ mice fed HCD following two i.v. injections of biotin-labeled lncRNA transcripts (*n* = 2 per group, pooled)**. g** RNA immunoprecipitation (RIP) in nuclear BMDM lysates using HuR-specific antibody (*n* = 3). **h** BMDM treatment with camptothecin (CPT) induces HuR (arrow head) and *MAARS (*arrow) shuttling from the nucleus to the cytosol. Representative images and quantification of cytosolic and nuclear HuR and *MAARS* staining in BMDM treated with or without camptothecin (CPT, 20 µM) for 1 h (*n* = 3, 200 cells quantified per condition). **i** HuR cytosolic (white arrows) and nuclear staining was quantified in confocal microscopy images of BMDMs transfected with control or *MAARS* gapmeRs (*n* = 3, 100 cells quantified per condition). **j** Quantification of Western blots of HuR in whole cell lysates from BMDMs transfected with control or *MAARS* gapmeRs treated with or without CPT (20 µM, 1 h). Expression of HuR in cytosolic (**k**) and nuclear fractions (**l**) from BMDMs transfected with control or *MAARS* gapmeRs treated with or without CPT (20 µM, 1 h) (*n* = 6, values are mean ± SD). For all panels values are mean ± SD. *p < 0.05; **p < 0.01; ***p < 0.001; *****p < 0.00001.

Because lncRNAs typically exert effects in cis or in trans, we first explored the possibility that *MAARS* knockdown might alter neighboring genes. However, we found no impact of MAARS knockdown for regulation of its neighboring genes (Supplementary Fig. 1j), suggesting a potential trans mechanism. To identify potential *MAARS*-interacting proteins that may inform mechanisms underlying the decreased progression of atherosclerosis in *MAARS*-deficient LDLR^−/−^ mice, biotin-labeled T7 in vitro transcribed *MAARS* or LacZ were incubated with nuclear protein lysates of RAW264.7 macrophages (Fig. 4a). Peptides that specifically bound to biotin-labeled *MAARS* transcript were identified by liquid chromatography-mass spectrometry (LC-MS/MS) analysis, capturing HuR (Hu antigen R, also known as ELAVL1, Embryonic Lethal, Abnormal Vision, Drosophila-Like 1) as a *MAARS*-binding protein (Fig. 4b and Supplementary Fig. 5a). The HuR protein was only detectable in the eluate of biotin-labeled *MAARS* as well as in the input BMDM nuclear protein or whole cells lysates (Supplementary Fig. 5b) compared to the LacZ negative control (*n* = 3). Similar HuR pulldown results were observed using 3-end biotinylated *MAARS* (Supplementary Fig. 5c). As HuR is an RNA-binding protein that interacts with specific ARE we identified 14 HuR-specific AREs in the *MAARS* transcript (Table 1)^23–26^. To investigate whether HuR is interacting with *MAARS* by binding to specific ARE motifs, we mutated these AREs and performed RNA pulldown (Fig. 4c, d). Remarkably, the HuR pulldown was dramatically decreased by 81% when nuclear protein lysates were incubated with ARE-mutated *MAARS*, as compared to the wild type (WT) *MAARS* transcript (Fig. 4d). To further confirm the ARE-specific binding of *MAARS* to HuR, we performed competition pulldown studies using biotinylated *MAARS* and ARE synthetic RNA oligonucleotides and observed a concentration-dependent inhibition of *MAARS* binding to HuR starting with 0.5:1 picomole ratio of ARE oligos to biotinylated *MAARS* (Fig. 4e). Furthermore, the ARE-specific binding of *MAARS* to HuR was confirmed in vivo after i.v. injection of biotin-labeled AREs-mutated and WT *MAARS*: HuR was recovered in aortic protein lysates of biotin-labeled WT *MAARS* injected ApoE^−/−^ mice fed HCD for 12 weeks, while it was absent in the ARE-mutated *MAARS* group (Fig. 4f). Successful delivery of biotin-labeled *MAARS* was verified by RT-qPCR (Supplementary Fig. 5d). In reverse-pulldown experiments, *MAARS* expression was increased by fivefold in RNA isolated after HuR immunoprecipitation (IP) compared to IgG control; this was specific to *MAARS*, compared to other nuclear transcripts such as Histone 3 (Fig. 4g). These data indicate that *MAARS* interacts with HuR both in vitro and in vivo.

**Table 1:** AU-rich elements identified in the *MAARS* transcript and the respective mutations in the AREmut biotinylated *MAARS* transcript used for RNA pulldown in Fig. 4d–f.

AU-rich elements	AU-rich mutations
TTTTTGT	TGCGCGT
AATTTTA	ACTCGCG
TTTTA	GCGTA
TTTTTAA	GCTGCA
TTTATTT	GCTAGCG
TTTTTGTTT	GCGCTGTGC
TTTGTTT	TCTGAGC
TTTAATT	CGCAGTC
AATATTT	CGTAGC
ATTTTC	AGCTGC
CTTTTAT	CTGCACT
ATTTA	AGCTA
TATTTATT	CAGCTGC
TTTCCTT	GCACCGG

**Fig. 5:**
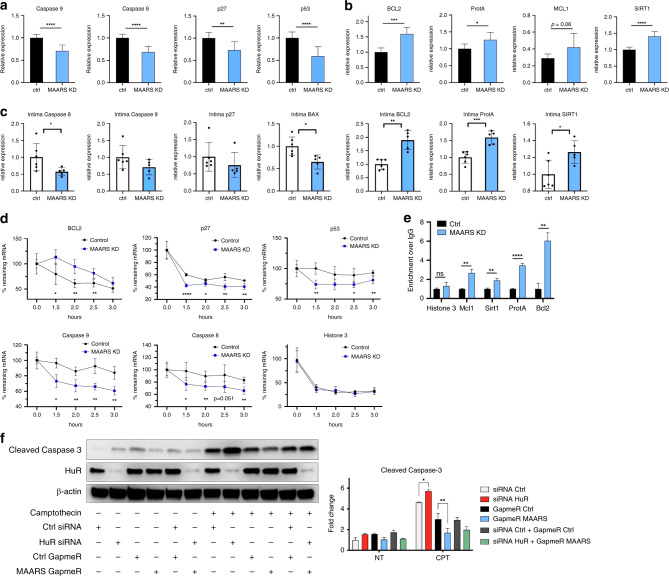
*MAARS* knockdown regulates HuR apoptotic targets. **a** *MAARS* knockdown decrease pro-apoptosis specific HuR targets in BMDMs transfected with *MAARS* gapmeRs (*n* = 4). **b** *MAARS* knockdown increases anti-apoptosis-specific HuR targets in BMDMs transfected with *MAARS* gapmeRs (*n* = 4). **c** Relative expression of HuR targets in the intima of LDLR^−/−^ mice treated with control (*n* = 6) and *MAARS* gapmeR (*n* = 5) (from study in Fig. 2). **d** mRNA stability studies of HuR targets in BMDMs transfected with control or *MAARS* gapmeRs and treated with camptothecin (CPT) for 1 h, then activated actinomycin D (100 ng ml^−1^) for the indicated time points (representative experiment of three independent experiments). **e** RNA immunoprecipitation (RIP) using HuR-specific antibody in lysates from BMDMs treated with *MAARS* or control gapmeR (shown is one RIP representative of three independent experiments). **f** Dependency studies of *MAARS* and HuR using the indicated gapmeRs and siRNAs, respectively, on cleaved caspase-3 expression in BMDMs treated with or without 20 µM CPT for 2 h. For all panels values are mean ± SD. *p < 0.05; **p < 0.01; ***p < 0.001; ****p < 0.0001.

A range of apoptotic stimuli (e.g., staurosporine, doxorubicin, prostaglandins) regulate HuR shuttling from the nucleus to the cytosol^27–30^. Here, we observed that in macrophages stimulated with CPT, *MAARS* shuttled together with HuR from the nucleus to the cytosol (Fig. 4h). Additionally, confocal microscopy and fractionation studies highlighted a cytosolic accumulation of HuR after *MAARS* knockdown using different gapmeRs (Fig. 4i–l and Supplementary Fig. 6a). Moreover, rescue experiments showed decreased HuR cytosolic shuttling after *MAARS* WT overexpression using lentivirus while lenti-AREmut had no significant effect (Supplementary Fig. 6b). While HuR silencing did not affect *MAARS* intracellular localization (Supplementary Fig. 6c), we propose a *MAARS* tethering mechanism of HuR in the nucleus. This interaction did not involve transcriptional or translational regulation of HuR, since *MAARS* silencing did not affect HuR expression at the mRNA level (Supplementary Fig. 6d, e) or in whole lysates of BMDMs (Fig. 4j). However, after *MAARS* knockdown we observed increased HuR expression in the cytosolic fraction and decreased expression in the nuclear fraction of BMDMs treated with or without CPT, suggesting that *MAARS* silencing regulates the HuR cytosolic shuttling (Fig. 4k, l). Moreover, HuR shuttling in the cytosol in response to *MAARS* gapmeR was concentration-dependent,(Supplementary Fig. 6f). HuR is an important RNA-binding protein that regulates apoptosis by binding to the mRNA of pro- and anti-apoptotic genes and mediating their stability or trafficking to the cytosol, among other mechanisms^12,16^. Importantly, *MAARS* knockdown regulated several well-described mRNA targets of HuR, decreasing the expression of p53, p27, and Caspase-8 and -9 in BMDMs (Fig. 5a) and of p27, Caspase-8, -9, and BAX in the intima of LDLR^−/−^ mice (Fig. 5c). In contrast, *MAARS* silencing increased the expression of anti-apoptotic genes BCL2, Prothymosin A (ProtA), Mcl-1, and SIRT1 in BMDMs (Fig. 5b) and of BCL2, ProtA, and SIRT1 in the intima of LDLR^−/−^ mice (Fig. 5c). Similar trends were observed in the spleens of LDLR^−/−^ mice after *MAARS* knockdown (Supplementary Fig. 6h), while no effects were observed in the aortic media fraction (Supplementary Fig. 6g). Consistently, MAARS silencing strongly regulated the mRNA stability of HuR targets, decreasing p53, caspase-8, -9, and p27, and increasing BCL2 after activation with CPT and treatment with the transcriptional inhibitor Actinomycin D (Fig. 5d). The mRNA stability of Histone 3, which is not a known HuR target, was not affected by *MAARS* knockdown, demonstrating specificity for HuR targets (Fig. 5d). RNA IP experiments in lysates from BMDM treated with *MAARS* or control gapmeRs confirmed the HuR-dependent effect on selected apoptosis genes: MAARS knockdown increased the HuR binding to Mcl1, SIRT1, ProtA, and Bcl-2 mRNAs, suggesting a potential MAARS buffering effect on HuR (Fig. 5e). Moreover, in MAARS knockdown BMDMs, lentiviral overexpression of WT MAARS rescued HuR binding to its targets by RIP in comparison to MAARS AREmut lentiviral overexpression (Supplementary Fig. 6i). In addition, dependency studies of silencing both HuR and *MAARS* showed that the increase in cleaved caspase-3 observed after HuR knockdown was abrogated in the presence of *MAARS* deficiency (Fig. 5f). Interestingly, although CPT and TNF-α decrease *MAARS* expression, potentially due to a feedback mechanism (Supplementary Fig. 5e, f), the MAARS binding affinity to HuR is not affected by CPT and TNF-α treatment in BMDMs as observed in the pulldown studies (Supplementary Fig. 5g). Taken together, these findings indicate that lncRNA *MAARS* actively binds the RNA-binding protein HuR, which in turn regulates the expression of a range of apoptosis genes in macrophages. Loss of *MAARS* in lesions thereby facilitates a protective anti-apoptotic program associated with regulation of mRNA stability of HuR targets and downstream decrease of caspase-8, -9, and -3 cleavage.

### *MAARS* knockdown regulates plaque necrosis and efferocytosis

**Fig. 6:**
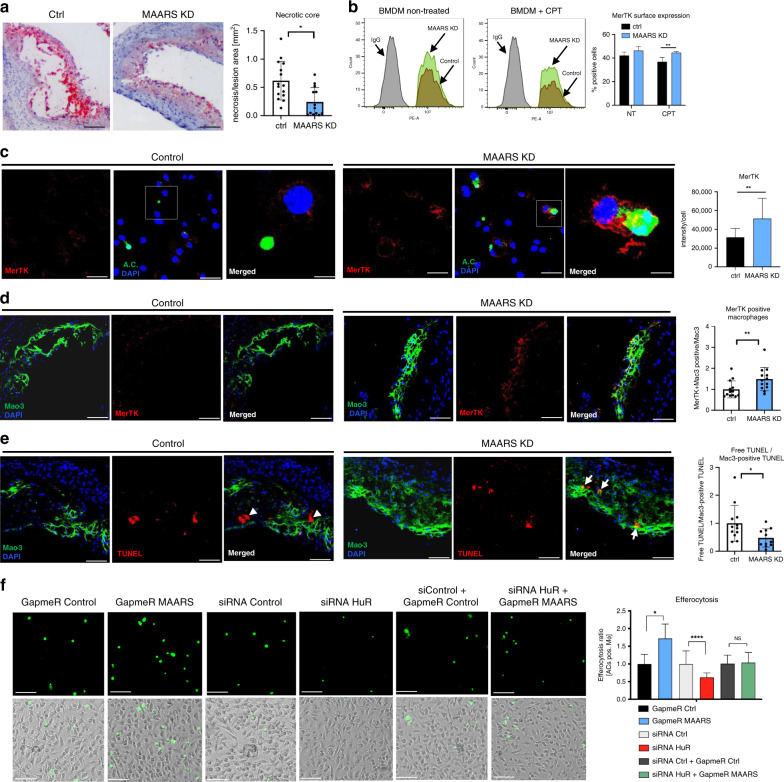
In vivo knockdown of *MAARS* inhibits plaque necrosis and increases efferocytosis. **a** Quantification of plaque necrosis in the lesions of the aortic sinus of LDLR^−/−^ mice fed HCD treated with control (*n* = 15) or *MAARS* gapmers (knockdown (KD), *n* = 13). **b** Flow cytometry staining with the efferocytosis marker MerTK in BMDMs treated with or without 20 µM CPT for 2 h and incubated with Jurkat apoptotic cells for 1 h at a BMDM:AC ratio of 1:10. **c** High resolution confocal images and quantification of MerTK staining in BMDMs treated with 20 µM CPT for 2 h and incubated with Jurkat apoptotic cells (AC) for 1 h at a BMDM:AC ratio of 1:1. **d** Evaluation of macrophage efferocytosis in vivo by co-staining with MerTK and Mac-3, and quantification of the ratio between double-positive MerTK^+^/Mac3^+^ and Mac3 staining alone in the aortic sinus of LDLR^−/−^ mice fed HCD treated with control (*n* = 15) or *MAARS* gapmers (KD, *n* = 13). **e** Quantification of the ratio between free TUNEL staining (arrow heads) vs. Mac3-positive TUNEL staining (arrows), reflecting Mac3-positive efferocytotic macrophages, in the aortic sinus lesions LDLR^−/−^ mice fed HCD treated with control (*n* = 15) or *MAARS* gapmeRs (KD, *n* = 13). **f** In vitro efferocytosis assay: BMDMs were transfected with control or *MAARS* gapmeRs in the presence or absence of the indicated control or HuR siRNAs and subsequently incubated with calcein AM-labeled apoptotic Jurkat cells for 1 h. Efferocytosis was quantified as the ratio of calcein-positive (<3 µm) vs. calcein-negative macrophages (*n* = 3, quantification of at least 20 images per condition). For all panels, values are mean ± SD; *p < 0.05; **p < 0.01; ***p < 0.001; ****p < 0.0001; ****p < 0.00001.

Plaque necrosis has been linked to alterations in the presence of lesional apoptotic cells^3,6^. We observed a lower number of necrotic cores in LDLR^−/−^ mice treated with MAARS-specific gapmeRs (by 60%) compared to LDLR^−/−^ mice treated with gapmeR controls (Fig. 6a). Necrotic core formation is associated with defective efferocytosis, or the ability of macrophages to engulf and degrade apoptotic cells before they become necrotic^3,31^. Macrophage MerTK (c-Mer tyrosinekinase) serves as a cell surface receptor and signaling molecule mediating efferocytosis, and MerTK has a central role in promoting efferocytosis and decreasing necrotic core formation in atherosclerotic lesions^32^. MAARS silencing in BMDMs incubated with Jurkat apoptotic cells increased MerTK surface expression as observed by flow cytometry (Fig. 6b and Supplementary Fig. 7a) and confocal microscopy (Fig. 6c). Furthermore, lesional macrophages (co-stained with Mac3) of LDLR^−/−^ mice treated with MAARS gapmeRs had increased expression of MerTK (by 49%) compared to control gapmeR injected mice (Fig. 6d). In a different approach to evaluate in vivo macrophage efferocytosis, aortic roots were co-stained with TUNEL and macrophage marker Mac-3 and the ratio of free vs. macrophage-associated apoptotic cells was assessed as previously described^33^. This ratio was markedly decreased by 53% in the atherosclerotic lesions of MAARS-knockdown mice, indicating more efficient efferocytosis (Fig. 6e). To further assess the role MAARS in macrophage efferocytosis in vitro, we used lentiviral overexpression studies. BMDMs overexpressing MAARS decreased efferocytosis of calcein-stained apoptotic Jurkat cells (Supplementary Fig. 7b). Conversely, in CPT-treated BMDMs, MAARS knockdown using two different gapmeRs significantly increased efferocytosis (by 72%) while HuR silencing had the opposite effect (39% decrease). In addition, silencing both MAARS and HuR showed that the MAARS-mediated effect on efferocytosis is dependent upon HuR. (Fig. 6f and Supplementary Fig. 7c). Further assessments by FACS or Western Blot of other mediators of efferocytosis such as dynamin-related protein 1, CD47 or CD172 (Serpin-α) did not show any significant regulation after MAARS silencing in BMDMs (Supplementary Fig. 7d, e).

**Fig. 7:**
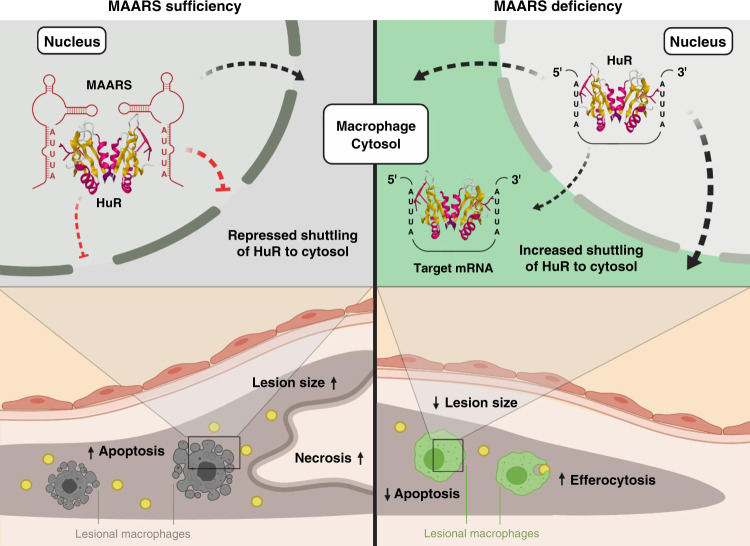
Proposed mechanism for *MAARS* regulation of HuR and effects on macrophages apoptosis, efferocytosis, and atherosclerosis. *MAARS* interacts with HuR (Protein Data Bank structure 6GC5) by tethering it in the macrophage nucleus, preventing its shuttling to the cytosol and interfering with its RNA-stabilizing function. *MAARS* deficiency induces HuR shuttling in the cytosol, decreasing macrophage apoptosis and increasing macrophage efferocytosis. Consequently, plaque necrosis and atherosclerosis progression is reduced. Created with BioRender.com.

To explore the pathways and biological processes affected by *MAARS* knockdown, unbiased genome-wide RNA-Seq was performed on *MAARS* gapmeR-transfected BMDMs. A number of dynamically regulated genes were identified (*p* < 0.0001) (Supplementary Fig. 8a). Network pathway analysis using MetaCore showed that the top up- and downregulated pathways were mostly related to apoptosis, inflammation, and efferocytosis: four apoptosis-related pathways were downregulated while pathways related to phagocytosis, a debris-cleaning process similar to efferocytosis, and cytoskeleton rearrangement pathways were upregulated (Supplementary Fig. 8a). Moreover, process network analysis of downregulated pathways indicated many apoptosis-related genes such as caspases, Bak, Bax, Bid, Bcl-XL, or cytochrome C (Supplementary Fig. 8b), while part of the upregulated genes in the network play roles in efferocytosis and cytoskeleton rearrangement, e.g., Profilin, Alpha-actin, Tubulin, ROCK, and Rho GTPases (Supplementary Fig. 8c). Taken together, these findings indicate that *MAARS* knockdown decreases macrophage apoptosis and increases their clearance, or efferocytotic capacity, thereby decreasing plaque necrosis in the atherosclerotic lesions.

## Discussion

Macrophage apoptosis occurs during all stages of atherosclerosis^34,35^. While some studies using different mouse models indicate that macrophage apoptosis in the early phases of atherosclerosis may be protective^36–40^, the accumulation of apoptotic debris in later stages, together with defective macrophage efferocytosis, may lead to the so-called “postapoptotic” or “secondary” necrosis and ultimately to atherosclerotic plaque progression^21^. Herein, we provide evidence that the macrophage-specific lncRNA *MAARS* regulates apoptosis by interacting with HuR, an RNA-binding protein that is a critical mediator of transcript stability and apoptosis. In support, lncRNA *MAARS* expression was potently induced in intimal lesions during the progression phase after 12 weeks of HCD in LDLR^−/−^ mice, a time point associated with a marked increase in lesional macrophage apoptosis and plaque necrosis along with reduced efferocytosis (Figs. 2f, g and 6a, d, e). Furthermore, neutralization of *MAARS* in the intima of lesions profoundly reduced plaque areas in the aortic sinus and thoracoabdominal aortas of LDLR^−/−^ mice (Fig. 2d, e). Importantly, these effects were predominantly independent of differences between groups in the lipoprotein profile (Supplementary Fig. 3b), lesional accumulation of leukocyte subsets (Supplementary Fig. 2), or inflammatory signaling pathways (Supplementary Fig. 4h), indicating that *MAARS* deficiency regulated a distinct pathway. Further interrogation of *MAARS*-deficient lesions revealed marked reduction in macrophage apoptosis and increased lesional efferocytosis. Mechanistically, *MAARS* deficiency reduced apoptotic markers such as p53, p27, and the caspase-3, -8, and -9 while increasing anti-apoptotic markers BCL2, Mcl1, and ProtA by interacting with HuR. In light of these findings, these studies identify *MAARS* as a potent regulator of HuR and macrophage apoptosis. In dependency studies, we show that HuR silencing induced apoptosis in macrophages, while *MAARS* knockdown can abrogate this phenotype as observed in the cleavage of caspase-3 (Fig. 5e).

This study highlights how cell-specific silencing of a lncRNA in the intima of atherosclerotic plaques using the nuclease-resistant LNA gapmeRs can exert powerful phenotypic switching. Other gapmeRs to lncRNAs have been recently described to regulate cardiac hypertrophy^41,42^. These studies demonstrated that delivery of gapmeRs can have cell-specific effects depending on the route of delivery and relative expression of interactors in target cells. In keeping with this, *MAARS* deficiency reduced macrophage apoptosis in the intima, but not in the media of aortic lesions (Fig. 2f and Supplementary Fig. 6e). As a consequence of macrophage apoptosis, cells may exhibit impaired homeostatic control of functions important to clearance of cellular debris or apoptotic cells^43^. Consistent with this notion, loss of *MAARS* in macrophages significantly reduced apoptotic markers and enhanced macrophage-mediated efferocytosis. These findings suggest that *MAARS*-deficient lesional macrophages are more functional, independent of lipid lowering or direct regulation of leukocyte recruitment to the vessel wall.

LncRNAs can exert their function by interacting with proteins, RNAs, and/or genomic DNA. Nuclear lncRNAs have been widely described to interact with heterogeneous nuclear ribonucleoproteins^44^. In this study, using RNA pulldown studies in combination with LC-MS/MS we have detected HuR/ELAVL1 as an interactor of *MAARS*. Our in vitro and in vivo data reveal a binding preference of *MAARS* to HuR, an RNA-binding protein and key regulator of cellular apoptosis^15,16^. The specific interaction of *MAARS* with HuR was confirmed using two pulldown methods in the BMDM nuclear fraction and in vivo after intravenous injection of biotinylated *MAARS* in ApoE^−/−^ mice fed HCD^9,10,45–47^. HuR is an RNA-binding protein that has been shown to control apoptosis by regulating mRNA stability, modulating translation, or by regulating mRNA nucleocytoplasmic shuttling^12,27^. Previous studies have documented a role of HuR in the stabilization of both pro-apoptotic genes such as p53, p27, caspase-8, caspase-9, cyclins A, B1, and D1^16,48–53^, and anti-apoptotic genes such as Bcl-2, Mcl-1, and Prothymosin α^12,54,55^, dependent on the stimuli, cell type, and stress intensity. In basal conditions, HuR is stabilizing the mRNA of anti-apoptotic genes such as Bcl-2, Mcl-1, and Prothymosine α,^12^ while HuR knockdown or knockout induces apoptosis^56,57^. However, under lethal stress HuR can have a pro-apoptotic phenotype after its cleavage, dependent on stimuli intensity^58^. Moreover, ATPase helicases regulate HuR ubiquitination and can induce an HuR-dependent destabilization of its mRNA targets such as p21^59^. While *MAARS* expression is increased with progression of atherosclerosis and decreased with regression in aortic macrophages (Fig. 1i), *MAARS* expression also decreased in BMDMs treated with TNF-α and CPT (Supplementary Fig. 5e, f). Several mechanisms can be responsible for the observed regulation in vitro, including compensatory or feedback mechanisms. Indeed, lncRNAs can serve as negative feedback regulators. One example is the lncRNA Mirt2 that is limiting the production of pro-inflammatory cytokines and protects mice from endotoxemia-induced fatality, but is itself increased in response to inflammatory stimuli^60^. While we have not identified a negative feedback mechanism for *MAARS*, we cannot rule out the existence of such mechanism in response to stress induced by apoptotic stimuli. Importantly, in all our experiments the CPT and TNF-α were added to the cells when the MAARS silencing was already achieved (~90%) in BMDMs (Supplementary Fig. 5e, f), hence the potential *MAARS* downregulation after the addition of CPT and TNF-α was likely negligible. Moreover, the *MAARS* binding affinity to HuR is not affected by CPT or TNF-α treatments in BMDMs, as observed in the pulldown experiments (Supplementary Fig. 5g).

Mechanistically, *MAARS* knockdown increased the expression of several well-described anti-apoptotic HuR mRNA targets (Bcl-2, SIRT1, Prothymosine α) in vitro and in vivo, while decreasing other pro-apoptotic HuR targets such as p53, p27 and the caspases-8 and -9, by regulating their mRNA stability (Fig. 5a–d). Furthermore, *MAARS* knockdown increased binding of HuR to stabilize the anti-apoptotic mRNAs Bcl-2, Mcl1, SIRT1, and Prothymosine α, suggesting that *MAARS* is buffering HuR in the nucleus (Fig. 5e). We show here that HuR interacts with *MAARS* by binding to specific ARE and mutating these sites in the *MAARS* transcript can dramatically decrease the *MAARS*–HuR interaction in vitro and in vivo (Fig. 4e, f). While *MAARS* tracks with HuR shuttling in the cytosol after apoptosis stimulation with CPT, *MAARS* knockdown increased the accumulation of HuR in the cytosol, suggesting that under basal conditions *MAARS* tethers HuR in the nucleus, preventing its trafficking to the cytosol, thereby preventing its functional role in mRNA stability (Fig. 4i, j).

Accumulating studies highlight that a reduction in macrophage apoptosis increases their efferocytosis potential and overall clearance of plaques debris, further reducing plaque necrosis^31,61,62^. We observed in this study that *MAARS* knockdown is associated with increased cell surface expression of MerTK, a key efferocytosis receptor, in vivo in the aortic intima and in BMDMs (Fig. 5a–d). Moreover, *MAARS* silencing increased the mRNA expression of SIRT1, an anti-apoptotic HuR target gene^49^ that is also known to increase macrophage efferocytosis^63,64^. However, besides its role in apoptosis and efferocytosis, SIRT1 is a key player in other cellular processes such as macrophage self-renewal^65^ or polarization^66,67^. Although we show here that *MAARS* knockdown strongly induced macrophage efferocytosis by increasing the expression of MerTK and of the HuR gene target SIRT1, there are likely alternative pathways involved in this process. In this study we show that *MAARS* deficiency in macrophages decreases apoptosis and increases their efferocytosis; consequently, this leads to lower plaque necrosis (Fig. 7). Taken together, these findings indicate that *MAARS* lncRNA can actively bind to the RNA-binding protein HuR and mediate apoptosis. These findings provide considerable insights and potential impact for controlling HuR and its nuclear-to-cytoplasmic shuttling that may be dysregulated in diverse disease states.

As *MAARS* knockdown mice still demonstrated a marked reduction in atherosclerotic lesion formation even after normalization of total cholesterol between groups (Supplementary Fig. 3b), we explored the possibility of a direct effect on cholesterol handling. In support, *MAARS* knockdown also influenced macrophage cholesterol metabolism, as it increased the cell cholesterol efflux to the main serum acceptors in BMDMs, (Supplementary Fig. 3c). Cholesterol efflux is involved in the regulation of total cell and membrane cholesterol content, both impacting on macrophage functions and atherosclerosis^68,69^. Thus, the impact of *MAARS* on macrophage cholesterol handling might be another one of the mechanisms of its proatherogenicity. In addition, as previous studies demonstrated that high cholesterol content can induce macrophage apoptosis^70–72^, future studies will be of interest to explore the relationship between the effect of MAARS on macrophage cholesterol metabolism and apoptosis.

In summary, we have identified lncRNA *MAARS* from intimal atherosclerotic lesions as a homeostatic regulator of macrophage apoptosis and efferocytosis by interaction with HuR. Deficiency of the lncRNA *MAARS* reduced lesional macrophage apoptosis leading to enhanced efferocytosis, and decreased atherosclerosis largely independent of lipid lowering, lesional leukocyte accumulation, or inflammatory signaling pathways. These findings demonstrate a role of this lncRNA as an interactor of an RNA-binding protein, HuR, to regulate macrophage apoptosis in the vessel wall. Strategies aimed at neutralizing *MAARS* expression or blocking *MAARS*–HuR interactions may provide a translational approach to limiting macrophage apoptosis and vascular remodeling in advanced plaques applicable to a range of chronic disease states.

## Methods

### Cell culture and transfection

Bone marrow was isolated from the femur and tibia of C57BL/6 mice and cultured in IMDM medium supplemented with 10 ng ml^−1^ mMCSF (mouse macrophage colony stimulation factor (416 ML, R&D), 10% FBS, and 1% Penicilin-Streptomycin. Medium was changed every 2 days and cells were used in experiments after at 7–10 days in culture. Transfection was performed using Lipofectamine 3000 (Invitrogen, 11668-019) as described in manufacturer’s protocol, and customized GapmeRs for *MAARS* (Qiagen, 25nmol except when mentioned differently) or negative control #1 (Qiagen). Cells were allowed to grow for 36 h before treatment with CPT (Sigma-Milipore) or TNF-α (210-TA/CF, R&D Systems) for 2 or 16 h, respectively. Cells were lysed in TRIzol (Invitrogen) or RIPA buffer (Boston BioProdcuts, BP-115) for further qPCR and Western Blot analysis, respectively^73,74^.

### RNA isolation and RT-qPCR

Tissues were homogenized using TissueLyser II (Qiagen) according to manufacturer’s instructions. For RNA isolation, TRIzol reagent (Invitrogen) or RNeasy kit (Qiagen) was used based on manufacturers protocol. Isolation of intimal RNA and subsequent RT-qPCR from aorta was performed as previously documented^73,74^. Briefly, aortas were carefully flushed with PBS, followed by intima peeling using TRIzol reagent (Invitrogen, 15596018). TRIzol was flushed for 10 s—10 s pause—another 10 s flushed and collected in an Eppendorf tube (~300–400 µl total) and snap frozen in liquid nitrogen. The intima specific isolation was assessed by qPCR showing enrichment of endothelial marker CD31 and macrophage marker Mac2 in the intima fraction compared to media/adventitia fraction (Supplementary Fig. 1c, d). Subsequent RT-qPCR was performed using High-Capacity cDNA Reverse Transcription kit (Applied Biosystems, 4368813). GoTaq qPCR Master Mix (Promega, A6001) was used for RT-qPCR experiments. Expression of mRNAs and lncRNA expression levels were normalized to GAPDH, HPRT, or β-actin (Aglient, AriaMx Real Time PCR System). Changes in expression were calculated using delta delta Ct method. Primer sequences are described in Table 2. *MAARS* copy number was quantified using the formula, as previously described^75,76^

$${\mathrm{number}}\,{\mathrm{of}}\,{\mathrm{copies}}\,{\mathrm{(molecules)}} = \frac{{X\;{\mathrm{ng}} \times {\mathrm{6}}{\mathrm{.0221}} \times {\mathrm{10}}^{{\mathrm{23}}} \, {\mathrm{molecules}}/{\mathrm{mole}}}}{{N \times {\mathrm{660}}\,{\mathrm{g}}/{\mathrm{mole}}^\dagger \times {\mathrm{1}} \times {\mathrm{10}}^{\mathrm{9}}\,{\mathrm{ng}}/{\mathrm{g}}}},$$

**Table 2:** Mouse-specific primers.

Primers	sequence
*MAARS* forward	TCACTTGTGCCCTGACTCTG
*MAARS* reverse	TCTCCAGCAACACAATCCAG
*MAARS* 5′RACE	GATTACGCCAAGCTTGGTAGGCTAAGGCTCAAAGCCTGGAACA
HuR forward	TTCTCGGTTTGGGCGAATCA
HuR reverse	ACTTCACTGTGATGGGCTCG
BCL2 forward	GAACTGGGGGAGGATTGTGG
BCL2 reverse	GCATGCTGGGGCCATATAGT
MCL1 forward	TGCTCCGGAAACTGGACATT
MCL1 reverse	TCCTGCCCCAGTTTGTTACG
Prothymosin A forward	CTCTCGCCAGAGTCCTCGAA
Prothymosin A reverse	GGAGCTGGTATCCACTGCC
Caspase-9 forward	TCCTGGTACATCGAGACCTTG
Caspase-9 reverse	AAGTCCCTTTCGCAGAAACAG
Caspase-8 forward	TGCTTGGACTACATCCCACAC
Caspase-8 reverse	TGCAGTCTAGGAAGTTGACCA
Caspase-3 forward	ATGGAGAACAACAAAACCTCAGT
Caspase-3 reverse	TTGCTCCCATGTATGGTCTTTAC
BAX forward	TGAAGACAGGGGCCTTTTTG
BAX reverse	AATTCGCCGGAGACACTCG
CDKN1B (p27) forward	TCAAACGTGAGAGTGTCTAACG
CDKN1B (p27) reverse	CCGGGCCGAAGAGATTTCTG
p53 forward	GTCACAGCACATGACGGAGG
p53 reverse	TCTTCCAGATGCTCGGGATAC
IL-1B forward	ATGCCACCTTTTGACAGTGATG
IL-1B reverse	AGCTTCTCCACAGCCACAAT
COX-2 forward	TTCAACACACTCTATCACTGGC
COX-2 reverse	AGAAGCGTTTGCGGTACTCAT
TNFa forward	CCCTCACACTCAGATCATCTTCT
TNFa reverse	GCTACGACGTGGGCTACAG
MCP-1 forward	TTAAAAACCTGGATCGGAACCAA
MCP-1 reverse	GCATTAGCTTCAGATTTACGGGT
Mac2 forward	GGAGAGGGAATGATGTTGCCT
Mac2 reverse	TCCTGCTTCGTGTTACACACA
GAPDH forward	AGGTCGGTGTGAACGGATTTG
GAPDH reverse	TGTAGACCATGTAGTTGAGGTCA
U6 forward	CTCGCTTCGGCAGCACA
U6 reverse	AACGCTTCACGAATTTGCGT
AREs synthetic RNA oligonucleotides	UCAUUAUUUAUUACGAUUUAUUUAUUAGCGAUUUAUUUAUUUACG

where Avogadro’s number = 6.02 × 10^23^ molecules/mole; *X* is the amount of amplicon (ng); *N* is the length of dsDNA amplicon; 660 g/mole = average mass of 1 bp RNA.

### 5′ RACE-PCR

5′ RACE-PCR (Rapid amplification of cDNA ends—Polymerase Chain Reaction) was performed based on the manufacturer’s protocol (ClonTech, 634858). Briefly, 0.5 µg total RNA from mouse BMDM was used and combined with 1 µl of gene-specific primer (10 µM stock) to a final volume of 11 µl. Tubes were incubated at 72 °C for 3 min, followed by 68 °C for 5 min and then placed immediately on ice. Protocol was followed as suggest by manufacturer. Primers were designed with Tm > 70 °C (Table 2). Following touchdown PCR, the product was loaded on 1% agarose gel and bands were isolated and cloned in vector for sequencing.

### Polyadenylation

RNA of 10^6^ BMDMs was isolated using TRIzol reagent (Invitrogen) and resuspended in RNase-free water. Polyadenylated and non-polyadenylated RNA was enriched with polyA Spin mRNA isolation kit (NEB, S1560S) based on manufacturer’s protocol. RT-PCR was performed with same input volume, independent of concentration and normalized to non-polyadenylated RNA fraction.

### Cellular fractionation

BMDMs fractionation for cytoplasmic and nuclear fractions was performed using the Active Motif fractionation kit (40010, Active Motif) according to the manufacturer’s protocol. The protein lysates concentration was determined and used for Western Blot. RNA was harvested as described previously and cleaned up using the RNeasy kit (Qiagen). Equivalent RNA volumes of cytoplasmic and nuclear associated RNA were converted to cDNA as described previously.

### Western blot

Proteins were isolated using RIPA buffer (Boston BioProdcuts, BP-115) with protease inhibitor and phosphatase inhibitors. Protein concentrations were determined using Pierce BCA assay (Thermo Scientific). In total, 20 μg protein were loaded per lane on a 4–20% Mini-PROTEAN TGX Gel (Bio-Rad, 456-1096). Separated proteins were transferred to PVDF membranes using the Transfer Turbo Blot system (Bio-Rad) and Trans-Blot Turbo RTA Transfer Kit (Bio-Rad, 170-4272). The membrane was blocked with 5% nonfat milk in TBST for 1 h at room temperature. After blocking, the membrane was incubated overnight at 4 °C with antibodies against Flag Tag (Cell Signaling, 2368, 1:1000), GAPDH (Cell Signaling, 2118, 1:4000), β-actin (Cell Signaling, #4970, 1:3000), cleaved Caspase-3 (Cell Signaling, 1:1000), cleaved Caspase-8 (Cell Signaling, 1:2000), HuR (12582S, Cell Signaling, 1:3000), Quantification of protein bands were performed using a luminescent image analyzer (Bio-Rad, Chemidoc).

### Protein-coding potential

In silico CPAT online algorithm was used for prediction of coding potential^77^. Transcripts for *MAARS* (Ensemble ID# ENSMUST00000067618.4) were synthesized by Genewiz. For in vitro validation of peptide coding potential, *MAARS* transcript was cloned upstream of p3xFLAG-CMV-14 expression vector (Sigma, E7908) using EcoRI restriction site. In total, 293 T cells were transfected with 500 ng plasmid using Lipofectamine 2000 (Invitrogen) and protein lysate was isolated 72 h post-transfection, followed by immunoblotting for FLAG Tag (Cell Signaling, 8146).

### RNA-ISH

Customized probe for *MAARS* was specifically developed to detect ENSMUST00000067618.4 (Advanced Cell Diagnostics). BMDMs were fixed in 4% paraformaldehyde and the ISH protocol for cultured adherent cells was performed as described by the manufacturer (RNAscope 2.5 HD Reagent Kit-Red; Advanced Cell Diagnostics, 322350).

### Apoptosis assays

TUNEL (Roche, 12156792910) and ApoLive-Glo Cleaved Caspase-3/7 (Promega G6410) assays were performed based on manufacturer’s protocol. Briefly, BMDMs were seeded in 96-well plate (Corning, 3610) and transfected with gapmeRs. After 36 h the cells were activated with CPT (20 μM) at indicated concentrations for 2 h and the reagents for each assay were added according to the protocols.

### Efferocytosis

Efferocytosis assay was performed as described in ref. ^78^. Briefly, 5 × 10^6^ cells/ml Jurkat cells were labeled with 5 µM AM Calcein (Invitrogen, LS-H2452-50). After 2 h incubation, cells were washed and irradiated with UV (150 mJ/cm^2^) with an open lid, followed by another 2 h incubation before apoptotic cells were added in a 1:1 ratio to gapmeR-transfected or lentivirus-transduced primary macrophages. After several rounds of gentle washing, macrophages were counted positive for internalized green apoptotic bodies if they contained >3 µm clusters of green dots. Quantification was performed from four images with a total of 400 macrophages. For quantification of in vivo efferocytosis, macrophages were stained using rat anti-Mac3 (BD Pharmingen, 553322, 1:900), as described below for immunofluorescence. TUNEL protocol was performed based on the manufacturer’s protocol (Roche, In situ cell death detection kit, TMR red) and the ratio of macrophage-free TUNEL over macrophage-associated TUNEL signaling was calculated^79^.

### Lentivirus production and transduction

Lentivirus for pUltra (Malcolm Moore, Addgene, 24129) were generated by co-transfection of 293 T cells (ATCC CRL-3216) using Lipofectamine 3000 (Life Technologies) with pMD2.G (Didier Trono, Addgene, 12259) and psPAX2 (Didier Trono, Addgene, 12260) in a ratio 3:2:1, respectively. Transfection mix was added dropwise to dish and medium was changed after 8 h. The supernatant was collected 2 days later by filtering through 0.45 µm filter and stored at −80 °C. Transduction of BMDM was carried out in 6-well or 12-well plate adding 1 ml lentiviral supernatant to 1 ml medium in combination with 8 µg/ml polybrene (American Bio, AB01643). Normal growth medium was replaced after 36 h.

### Cholesterol efflux determination

For cholesterol efflux determination, BMDMs were labeled with 2 µCi/ml [1,2-^3^H]-cholesterol (Perkin Elmer, Waltham, MA, USA) for 24 h in the presence of an acyl-CoA:cholesterol acyltransferase inhibitor (Sandoz 58035, 2 µg/ml; Sigma-Aldrich, Milano, Italy) and 25 µg/ml acetylated LDL isolated and chemically modified from healthy donors by ultracentrifugation^80,81^. BMDMs were then incubated with 0.2% free fatty acid bovine serum albumin (BSA; Sigma-Aldrich, Milano, Italy) in IMDM for 18 h and then treated for 6 h with 10 µg/ml lipid free human apolipoprotein AI (Sigma-Aldrich, Milano, Italy), 12,5 µg/ml HDL (isolated from healthy donors by ultracentrifugation^81^, or 2% (v/v) human serum (NS) obtained from a pool of normolipidemic subjects, as cholesterol acceptors. Cholesterol efflux was expressed as a percentage of [1,2-^3^H]-cholesterol released into the medium over the total radioactivity incorporated by cells.

### LncRNA pulldown and liquid chromatography-mass spectrometry (LC-MS/MS)

LncRNA pulldown and LC-MS/MS was performed as previously documente^45^. For lncRNA pulldown, biotinylated RNA was generated using T7 RNA polymerase (Promega, P1300) by adding 1 µg linearized plasmid DNA, Biotin RNA labeling mix (Roche, 11685597910), 10× transcription buffer (Promega, P1300) and related RNase-free water in a total volume of 20 μl. The mix was incubated at 37 °C for 2 h. After incubation, 2 μl Dnase I (NEB, M0303L) was added and incubated at 37 °C for another 15 min to remove DNA template and the reaction was stopped by adding 0.8 μl 0.5 M EDTA (pH 8.0). Biotinylated RNA purification was performed using G-50 Sephadex Columns (Roche, 11273965001) according to the manufacturer’s protocol. After determination of the RNA concentration, the purified biotinylated RNA was immediately used or stored at −80 °C. Ten pmol of biotinylated RNA was heated for 2 min at 90 °C in RNA structure buffer (10 mM Tris pH 7.0, 0.1 M KCl, 10 mM MgCl2). After incubation, immediately transfer the mix on ice and incubate for another 2 min and incubated at room temperature for 20 min. Nuclear pellets were prepared by resuspending pellet of 107 HUVECs in 2 ml ice-cold PBS, 2 ml ice-cold unclear isolation buffer (1.28 M sucrose, 40 mM Tris-HCl pH 7.4, 20 mM MgCl2, 4% Triton X-100) and 6 ml ice-cold RNase-free water and incubated on ice for 20 min. After incubation, nuclei pellets were harvested by centrifugation at 2500 × *g* for 15 min, nuclear pellets were then resuspended in 1 ml RIP buffer (0.15 M KCl, 25 mM Tris-HCl pH 7.4, 5 mM EDTA, 0.5% NP-40, 0.5 mM DTT (Sigma, 646563), 100 U/ml RNase inhibitor (Invitrogen, AM2684), 1x protease inhibitor cocktail). Nuclei were homogenized by 18 strokes using a dounce homogenizer, followed by centrifuging for 15 min at 15,000 × *g*. The supernatant containing nuclear protein was transferred to a new tube and precleared by applying 60 μl of Streptavidin agarose beads (Thermo Scientific, 20347) for 1 h at 4 °C. Ten pmol properly folded biotinylated RNA and 1 μg/μl yeast tRNA (Ambion, AM7119) were added into the precleared nuclear lysate (200 μg) and incubated for 2 h at 4 °C, followed by addition of 60 μl of Steptavidin agarose beads and incubation for 1 h at 4 °C. At the end of the incubation, beads were collected by centrifugation at 12,000 × *g* and washed with 1 ml ice-cold NT2 buffer (50 mM Tris-HCl pH 7.4, 0.15 M NaCl, 1 mM MgCl2, 0.05% NP-40, 100 U/ml RNase inhibitor, 400 nM Vanadyl-ribonucleoside complex (BioLabs, S1402S), 1x protease inhibitor cocktail) at 4 °C five times. After washing, proteins were denaturated in 40 μl 2×Laemmli loading buffer (4%SDS, 120 mM Tris-HCl pH 6.8, 0.02% bromophenol blue, 0.2 M DTT) at 98 °C for 8 min for subsequent immunoblotting or resuspended in wash buffer without NP-40 for mass spectrometry analysis. For in vivo pulldown, biotinylated RNA was injected on two constitutive days by tail vein (15 µg/injection) before aortas were isolated on day 3. The tissue was processed as described above for cell lysate.

### Liquid chromatography-mass spectrometry (LC-MS/MS)

LC-MS/MS was performed as previously described^82^. Briefly, lncRNA pulldown of *MAARS* or LacZ purified samples were reduced with 10 mM DTT for 30 min at 56 °C in the presence of 0.1% RapiGest SF (Waters). Cysteines were alkylated with 22.5 mM iodoacetamide for 20 min at room temperature in the dark. Samples were digested overnight at 37 °C with trypsin. Rapigest was then cleaved according to manufacturer’s instructions and peptides purified by reversed phase and strong cation exchange chromatography. Peptides were loaded onto a precolumn (4 cm POROS 10R2, Applied Biosystems), resolved on a self-packed analytical column (12 cm Monitor C18, Column Engineering) after gradient elution (NanoAcquity UPLC system, Waters; 5–35% B in 90 min; *A* = 0.2 M acetic acid in water, *B* = 0.2 M acetic acid in acetonitrile), and introduced to the MS (TripleTOF 5600, ABSciex, Framingham, MA) by ESI (spray voltage = 2.2 kV). The mass spectrometer was programmed to perform data-dependent MS/MS (unit resolution, *m*/*z* 100–2000) on the 20 most abundant precursors in each MS1 scan (*m*/*z* 300–2000; accumulation time = 0.5 s; threshold = 70 counts; charge state = 2+ to 5+) using a rolling collision energy. After MS/MS, each precursor was excluded for 25 s. Raw data were converted to.mgf using ABSciex MSDataConverter; precursor and product ions were recalibrated using a linear equation derived from fitting experimentally observed masses obtained in an initial low mass tolerance database search. Recalibrated data were matched to peptide sequences in a forward/reversed human NCBI refseq database using Mascot version 2.4.1. Search parameters included trypsin specificity with up to two missed cleavages, fixed carbamidomethylation (C, +57 Da) and variable oxidation (M, +16 Da). Precursor and product mass tolerances were 12 ppm and 25 mmu. Protein hits from *MAARS*-specific pulldown were compared to negative control (LacZ) (*n* = 2, with two technical replicates).

### Animal studies

All protocols concerning animal use were approved by the Institutional Animal Care and Use Committee at Brigham and Women’s Hospital and Harvard Medical School, Boston, MA and conducted in accordance with the National Institutes of Health Guide for the Care and Use of Laboratory Animals. Studies were performed in LDLR^−/−^ mice (Jackson Laboratory, Stock#: 002207) or in C57Bl/6 mice (Charles River, Strain code#027).

### Immunohistology and characterization of atherosclerotic lesions

To quantify atherosclerosis in LDLR^−/−^ mice that were placed on HCD (Research Diets Inc., D12108C), aortic roots and aortic arch were embedded in OCT and frozen at −80 °C. Serial cryostat sections (6 µm) were prepared using tissue processor Leica CM3050. For lesion characterizations, the thoracic-abdominal aorta and aortic root were stained for ORO, macrophages (anti-Mac3, BD Pharmingen, 553322, 1:900) T cells (anti-CD4, BD Pharmingen, 553043, 1:90; anti-CD8, Chemicon, CBL1318, 1:100), MHC-positive cells (anti-MHCII, Novus Biologicals), and vascular smooth muscle cells (SM-α-actin, Sigma, F-3777, 1:500)^73,83^. The staining area was measured using Image-Pro Plus software, Media Cybernetics, and CD4+ and CD8+ cells were counted manually.

### Immunofluorescence staining

For immunofluorescence staining, 6 µm frozen sections of aortic roots and aortic arches were fixed with cold-acetone or 4% paraformaldehyde for 5 and 15 min, respectively, and blocked in PBS containing 3% BSA for 1 h at room temperature. Sections were stained with rat anti-Mac2, (Cedarlane, CL8942AP, 1:100), anti-Mac3 (BD Pharmingen, 553322, 1:900), rabbit anti-cleaved caspase-3 (Cell Signaling, 9664, 1:400) rabbit anti-HuR/ELAVL1 (12582S Cell Signaling and AB242410, Abcam), biotin goat anti-MerTK (BAF591, R&D Technologies) in PBS at 4 °C overnight. Similarly, BMDMs were plated on microscope slides and after treatment were fixed in 4% paraformaldehyde, permeabilized with 0.1% Triton X-100, and incubated with selected antibodies overnight as above. After three washes with PBS, the sections were further incubated with Alexa Flour 488-conjugated donkey anti-Rat IgG (Invitrogen, A21208, 1:300) and Alexa Flour 555-conjugated goat anti-Rabbit IgG (Invitrogen, A21428, 1:300) and anti-streptavidin Alexa-488 (Life Technologies) in PBS for 1 h at room temperature. Nuclei were stained for 5 min at room temperature in PBS containing DAPI (Cell signaling, 4083, 0.5 µg/ml). Coverslips were mounted with ProLong Gold antifade reagent (Invitrogen, P36930). Images were acquired on an upright Carl Zeiss LSM 510 confocal. TUNEL protocol was performed as described by manufacturer’s protocol (In Situ Cell Death Detection Kit TMR red, 12156792910, Roche).

### Lipid profile analysis

Lipid profile was measured as follows: the triglycerides were determined using InfinityTM Triglycerides Liquid Stable Reagent (Thermo Scientific). Total cholesterol was measured using the InfinityTM Cholesterol Reagent (Thermo Scientific) and HDL cholesterol was measured by colorimetric assay (BioAssay Systems, EnzyChromTM HDL). LDL cholesterol levels were calculated using the following formula: LDL = total cholesterol − HDL cholesterol − (triglycerides/5)^73^. Standards were purchased from Pointe Scientific, Inc.

### Liver function assays

AST (cat. No. EASTR-100) and ALT (cat. No. EALT-100) were detected using the BioAssay Systems kits according to the manufacturer’s protocol.

### RNA-Seq analysis

RNA-Seq analysis was performed after ribodepletion and standard library construction using Illumina HiSeq2500 V4 2 × 100 PE (Genewiz, South Plainfield, NJ). All samples were processed using an RNA-seq pipeline implemented in the bcbio-nextgen project (https://bcbio-nextgen.readthedocs.org/en/latest/)^17^. Raw reads were examined for quality issues using FastQC (http://www.bioinformatics.babraham.ac.uk/projects/fastqc/) to ensure library generation and sequencing were suitable for further analysis. Trimmed reads were aligned to UCSC build mm10 of the Mouse genome, augmented with transcript information from Ensembl release 79 using STAR^84^. Alignments were checked for evenness of coverage, rRNA content, genomic context of alignments (for example, alignments in known transcripts and introns), complexity, and other quality checks using a combination of FastQC, Qualimap. Counts of reads aligning to known genes were generated by featureCounts^85^. Differential expression at the gene level were called with DESeq2. The total gene hit counts and CPM values were calculated for each gene and for downstream differential expression analysis between specified groups was performed using DESeq2 and an adapted DESeq2 algorithm, which excludes overlapping reads, called no-overlapping reads. Genes with adjusted FDR < 0.05 and log2-fold change (1.5) were called as differentially expressed genes for each comparison. Mean quality score of all samples was 35.67 within a range of 40,000,000–50,000,000 reads per sample. All samples had at least >70% of mapped fragments over total. “MetaCore (v20.2) was used for functional enrichment analysis.”

### Statistics

Data throughout the paper are expressed as mean ± SD. Statistical differences were calculated using unpaired two-tailed Student’s *t* test or one-way ANOVA with Bonferroni correction for multiple comparisons. A probability of *p* < 0.05 was considered statistically significant. Ns not significant; *p < 0.05; **p < 0.01; ***p < 0.001; ****p < 0.0001. For illustration of differentially expressed genes Prism GraphPad software (V.7.0a) was used.

### Reporting summary

Further information on research design is available in the Nature Research Reporting Summary linked to this article.

## Supplementary information

Supplementary Information

Reporting Summary

## Data Availability

All relevant data are available from the authors. The RNA-seq data are accessible at: https://www.ncbi.nlm.nih.gov/geo/query/acc.cgi?acc=GSE155842. Source data are provided with this paper.

## Source data

Source Data
