# Supplementary material for: A macrophage-specific lncRNA regulates apoptosis and atherosclerosis by tethering HuR in the nucleus
Source: Nat Commun. 2020 Dec 1;11:6135. doi: 10.1038/s41467-020-19664-2 (PMC7708640; doi:10.1038/s41467-020-19664-2)
Supplement: Supplementary file 1 — Supplementary Information [file 41467_2020_19664_MOESM1_ESM.pdf]

Supplementary Information

A macrophage-specific lncRNA regulates apoptosis and atherosclerosis by tethering HuR in the nucleus

(V. Simion et al.)

Description of Supplementary File:

Supplementary Figure 1. Identification and characterization of lncRNA *MAARS*.

Supplementary Figure 2. *MAARS* knockdown (KD) in LDLR-/- mice fed HCD does not significantly affect the cellular composition of atherosclerotic lesions.

Supplementary Figure 3. *MAARS* knockdown effects on lipid metabolism.

Supplementary Figure 4. Effects of *MAARS* gapmeRs on pro-inflammatory markers and signaling pathways.

Supplementary Figure 5. *MAARS* interacts with HuR and plays a role in apoptosis.

Supplementary Figure 6. *MAARS* regulates HuR shuttling and its apoptosis target genes

Supplementary Figure 7. *MAARS* regulates macrophage efferocytosis

Supplementary Figure 8. Unbiased RNAseq analysis of *MAARS* knockdown in BMDM

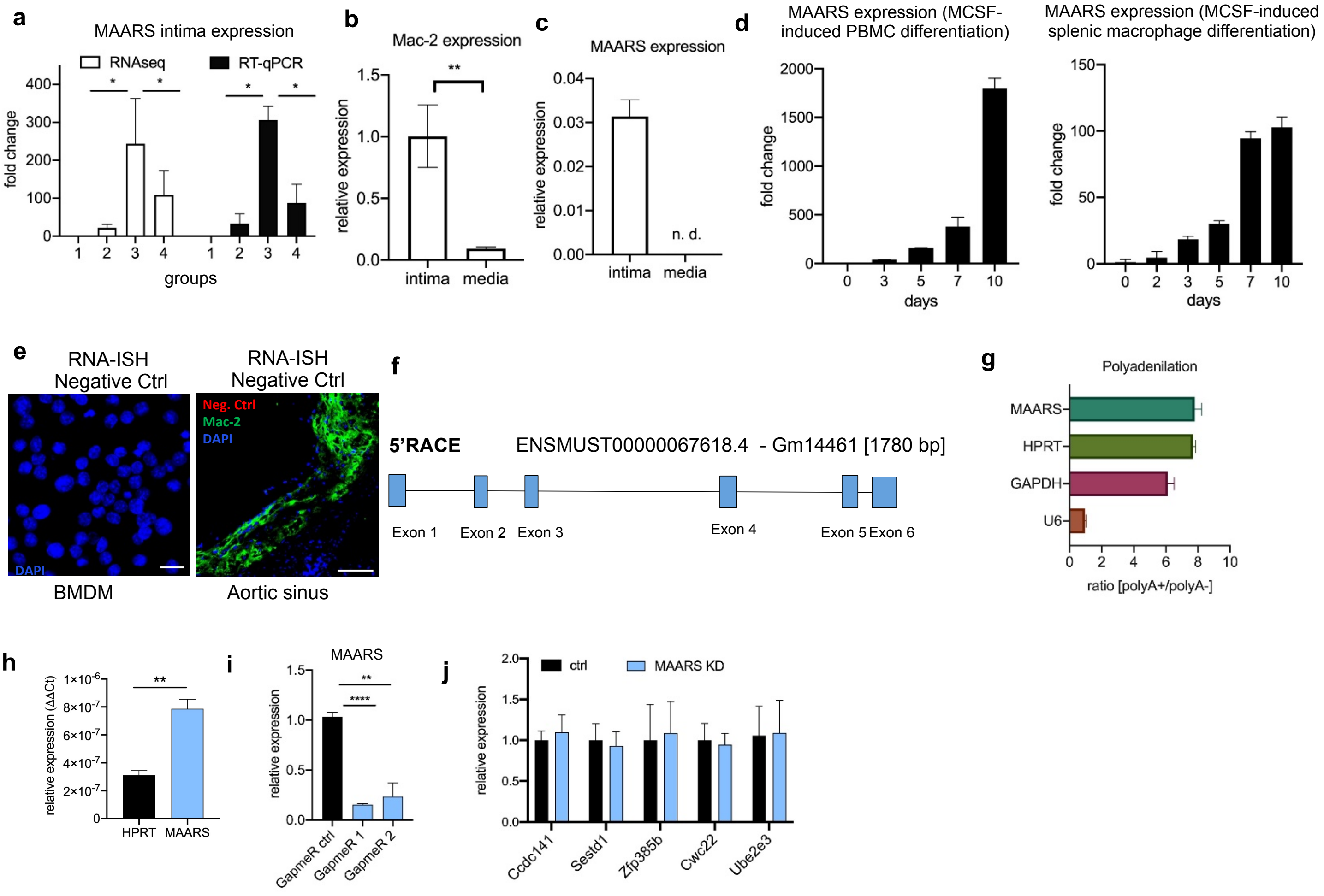

Supplementary Figure 1. Identification and characterization of lncRNA MAARS. Expression of macrophage marker Mac2 (**a**) and lncRNA *MAARS* (**b**) in the intima and media fractions obtained from the intima peeling protocol. **c** RNA-Seq results for *MAARS* across groups 1-4 obtained by RNAseq analysis and verified by RT-qPCR (n=3). **d** *MAARS* expression in PBMC differentiated macrophages (left panel) and in spleen-differentiated macrophages (right panel) for 10 days with macrophage colony-stimulating factor (MCSF). **e** Negative controls for RNA-*in situ* hybridization (ISH) in bone marrow-derived macrophages (BMDMs) and aortic sinus of LDLR$^{-/-}$ mice fed HCD. **f** 5'RACE-PCR for *MAARS* lncRNA in mouse from RNA of BMDM (n=3). **g** RNA from BMDMs was isolated for polyA+ and polyA-enriched RNA and analyzed by RT-qPCR. (n=3). **h** *MAARS* expression in BMDMs in comparison to HPRT housekeeping gene. **i** *MAARS* silencing efficiency using two different *MAARS* gapmeRs in BMDMs (n=3). **j** Effect of *MAARS* knockdown in BMDMs on expression of *MAARS* neighboring genes (n=3). For all panels, values are mean ± SD; *p < 0.05, **p<0.01; ***p<0.001.

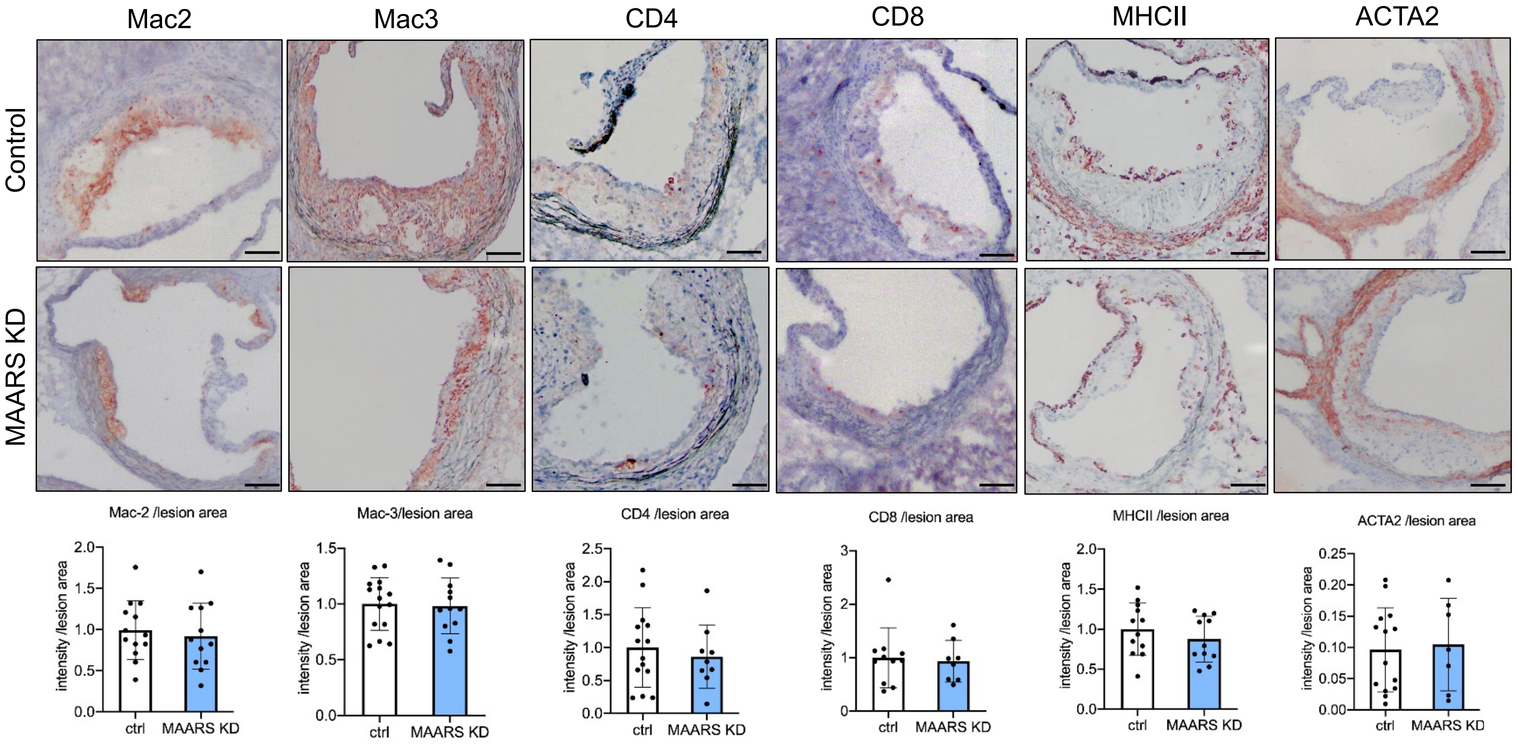

Supplementary Figure 2. *MAARS* knockdown (KD) in LDLR$^{-/-}$ mice fed HCD does not significantly affect the leukocyte composition of atherosclerotic lesions. Representative images and quantification for Mac-3, Mac-2, CD4, CD8, MHCII, and ACTA2 in lesions of the aortic sinus of LDLR$^{-/-}$ HCD mice treated with control (n=15) or *MAARS* (n=13) gapmeRs after 12 weeks. For all panels, values are mean ± SD.

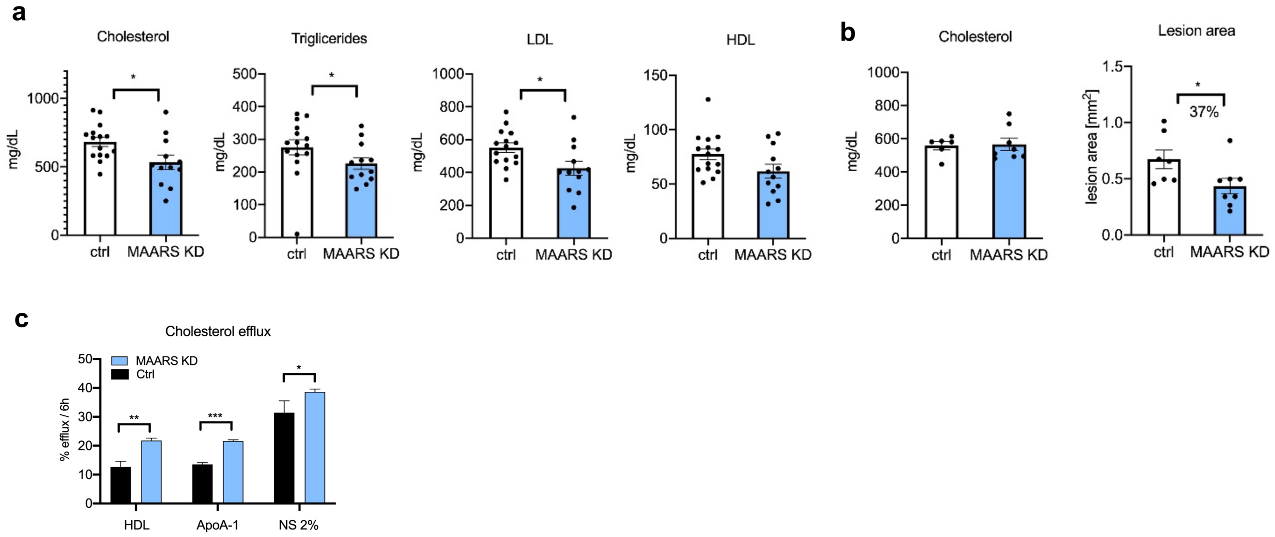

Supplementary Figure 3. ***MAARS knockdown effects on lipid metabolism*****. a** Circulating lipid levels (total cholesterol, Triglycerides, LDL-C, and HDL) in the $LDLR^{-/-}$ HCD mice treated with control (n=15) or *MAARS* (n=13) gapmeRs after 12 weeks. **b** When total cholesterol is normalized between $LDLR^{-/-}$ mice treated with control or *MAARS* gapmeRs, there is still a significant reduction of lesion area in the aortic sinus in the *MAARS* knockdown (KD) group. **c** Effect of *MAARS* knockdown on bone marrow derived macrophage (BMDM) cholesterol efflux using HDL, ApoA-1, or 2% normal serum (NS) as acceptors. For all panels, values are mean ± SD; *p < 0.05, **p<0.01; ***p<0.001.

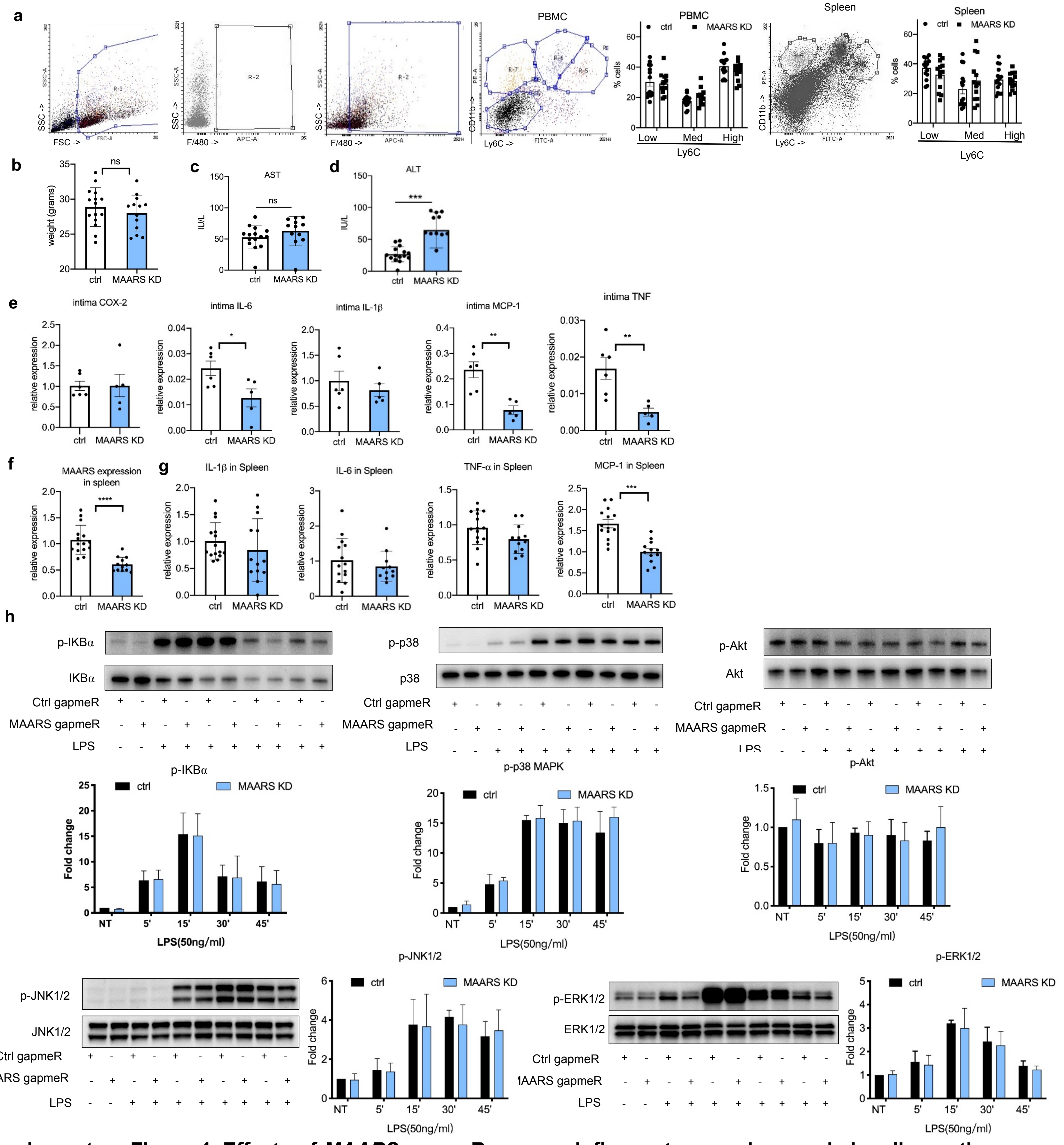

Supplementary Figure 4. Effects of *MAARS* gapmeRs on pro-inflammatory markers and signaling pathways. a Surface expression of Ly6C and CD11b in F4/80-sorted peripheral blood mononuclear cells (PBMCs) and splenic cells from LDLR$^{-/-}$ mice on HCD treated for 12 weeks with *MAARS* (n=13) or control gapmeRs (n=15). Relative expression of inflammatory genes in the intima of Control- and MAARS-KD LDLR$^{-/-}$ mice fed HCD. **b** Weight of MAARS- and control-injected mice. Serum levels of aspartate transaminase, AST (**c**), alanine transaminase, ALT (**d**) in mice injected with MAARS- and control-gapmeRs. Relative expression of *MAARS* (**f**) and inflammatory genes in the intima (**e**) or spleens (**g**) of control- and *MAARS*-KD LDLR$^{-/-}$ mice fed HCD. **h** There are no effects on *MAARS* silencing on NF-κB, P38K, JNK, ERK, or AKT inflammatory signaling pathways in bone marrow derived macrophages (BMDMs) treated with MAARS-specific or control gapmeRs. For all panels, values are mean ± SD; *p < 0.05, **p<0.01; ***p<0.001.

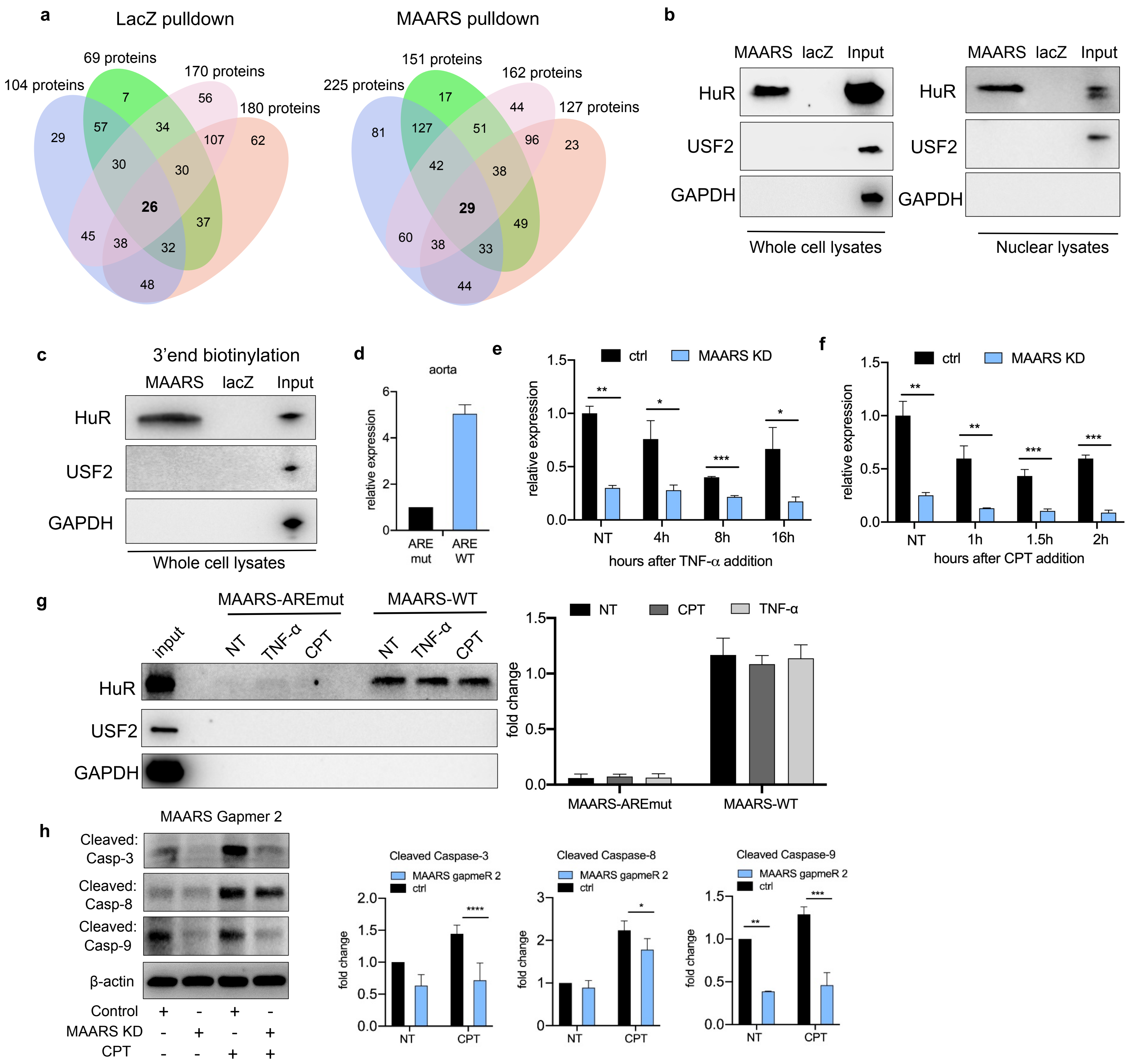

Supplementary Figure 5. *MAARS* interacts with HuR and plays a role in apoptosis. a Mass Spectrometry peptide hits for lacZ and MAARS (n=2, two technical replicates each, protein score cut off=2). **b** Immunoblotting for HuR, Histone 3, and GAPDH in nuclear protein lysates or HuR, GAPDH, and α-tubulin in whole cell lysates from RAW264.7 macrophages after lncRNA pulldown (representative images, n=3). **c** lncRNA pulldown using 3'end lncRNA biotinylation in nuclear lysates of RAW264.7 macrophages. **d** RT-qPCR analysis for intravenous delivery of biotinylated *MAARS* to the aorta of LDLR$^{-/-}$ mice. *MAARS* expression in BMDMs treated with TNF-α (**e**) or camptothecin, CPT (**f**) for selected time points. **g** Biotinylated lncRNA pulldown using *MAARS* wild type (WT) or *MAARS* with mutated HuR binding ARE sequences (AREmut) in lysates of BMDMs treated with CPT (20 μM) or TNF-α (50 ng/ml) (n=3). **h** *MAARS* knockdown using gapmeR-2 induces a similar anti-apoptotic phenotype as gapmeR-1 on expression of cleaved caspase-3, -8, and -9. For all panels, values are mean ± SD. *p < 0.05, **p<0.01; ***p<0.001.

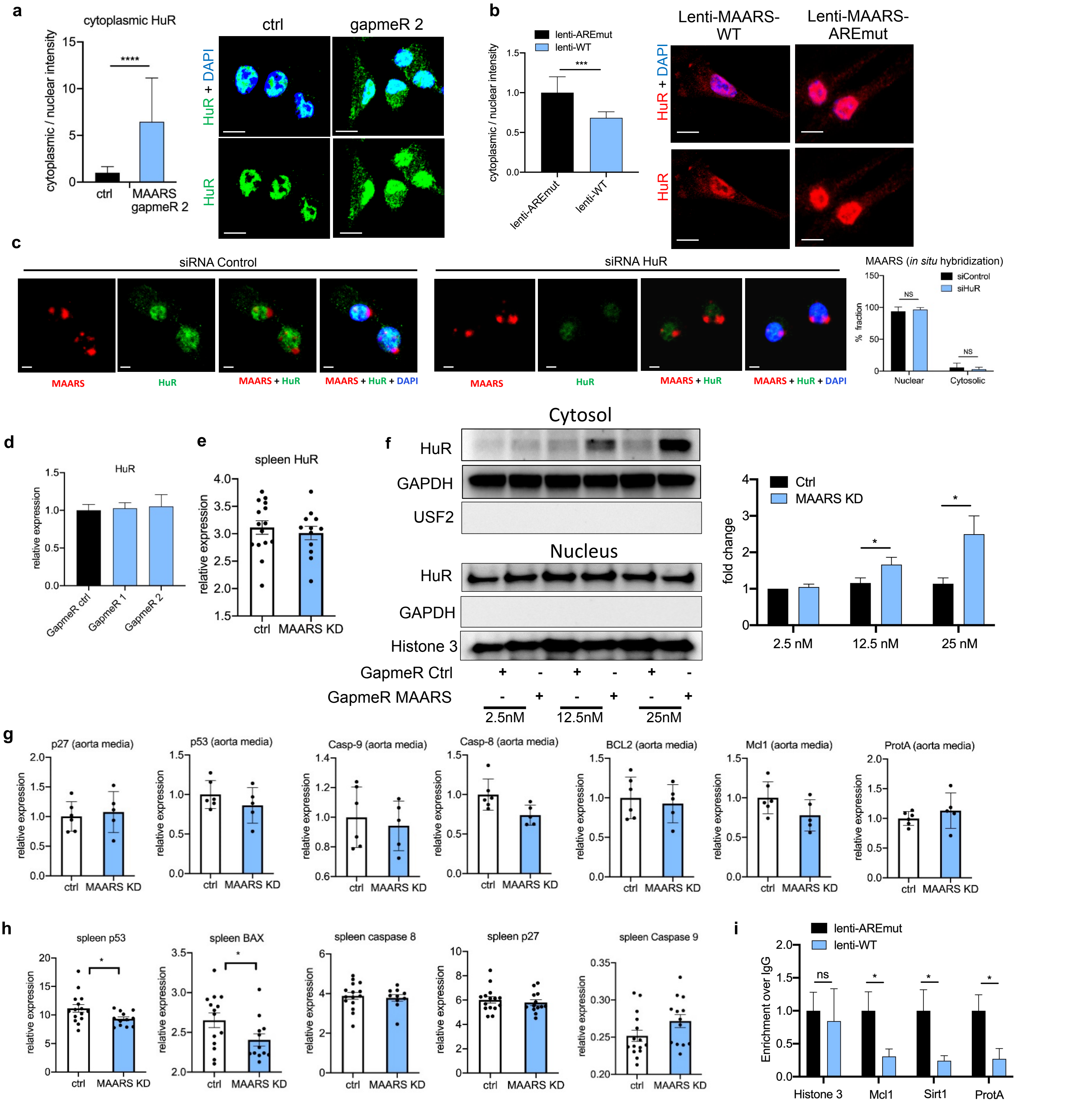

Supplementary Figure 6. *MAARS* regulates HuR shuttling and its apoptosis target genes. a *MAARS* knockdown using gapmeR-2 induces a similar HuR cytosolic shuttling as gapmeR-1 (representative of three independent experiments). **b** After MAARS knockdown in BMDMs, lentiviral overexpression of MAARS wild type (WT) reverses the HuR shuttling into the cytoplasm compared to the MAARS AREmut lentivirus. **c** Representative images and quantification of *MAARS* nuclear and cytosolic localization by RNA fluorescence in situ hybridization (FISH) after HuR silencing in BMDMs co-stained with HuR antibody. **d** Effect of MAARS silencing on HuR mRNA expression using two different *MAARS* gapmeRs transfected in BMDMs in vitro. **e** Effect of *MAARS* knockdown (KD) on HuR expression in spleens of LDLR$^{-/-}$ fed HCD treated with gapmeR control or *MAARS*-specific gapmeRs. **f** Western Blot detection of HuR, GAPDH, α-tubulin, and Histone 3 in cytosolic and nuclear fractions of BMDMs treated with increasing concentrations of control or *MAARS*-specific gapmeRs, n=3. **g** Relative expression of apoptotic genes in the media fraction of aorta from control and *MAARS* KD groups of LDLR$^{-/-}$ mice. **h** Relative expression of apoptotic genes in the spleens of control and *MAARS* KD groups of LDLR$^{-/-}$ mice. **i** RNA immunoprecipitation (RIP) using HuR-specific antibody in lysates from MAARS knockdown in BMDMs after lentivirus overexpression of MAARS wild type (WT) or MAARS AREmut (shown is one representative RIP of three independent experiments). For all panels, values are mean ± SD. *p < 0.05, **p<0.01; ***p<0.001.

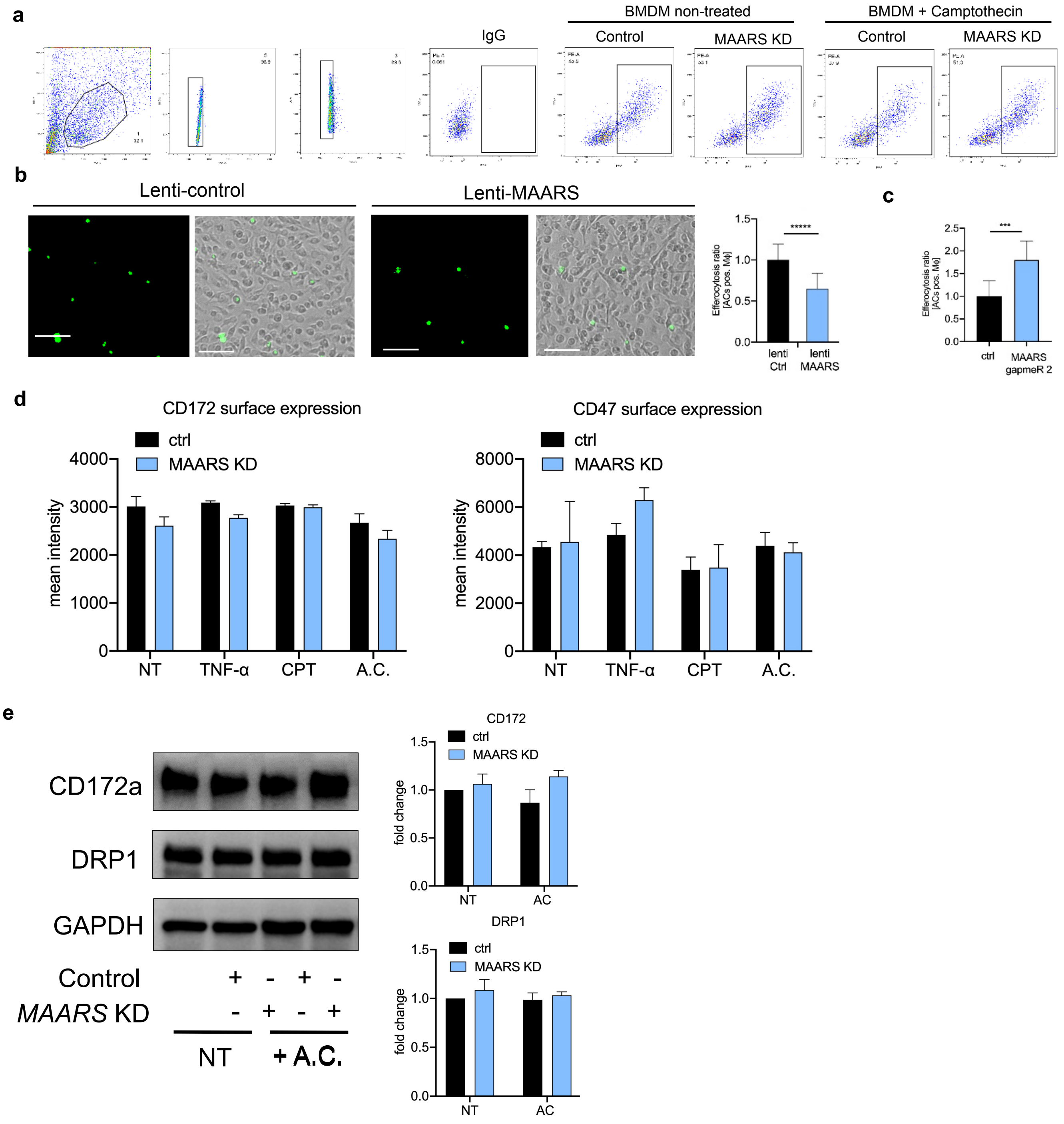

Supplementary Figure 7. *MAARS* regulates macrophage efferocytosis. a Representative FACS gating images of MerTK staining for histograms presented in Figure 6b. *In vitro* efferocytosis assay in BMDMs treated with control and MAARS gapmeR-2 (**b**) or transduced with control and *MAARS*-overexpressing lentivirus (**c**) and subsequently incubated with calcein AM-labeled apoptotic Jurkat cells for 1hr. Efferocytosis was quantified as the ratio of calcein-positive (<3μm) vs. calcein-negative macrophages (n=3, quantification of at least 20 images per condition). **d** Flow cytometry cell surface expression of CD172 and CD47 in MAARS knockdown BMDMs that were incubated with CPT (20 μM), TNF-α (50 ng/ml), or Jurkat apoptotic cells (A.C.), representative of three independent experiments. **e** Western blot analyses of CD172 or DRP1 expression from whole cell lysates of MAARS knockdown BMDMs incubated with Jurkat apoptotic cells (A.C.), n=3. For all panels, values are mean ± SD. *p<0.05, **p<0.01; ***p<0.001; ****p<0.0001.

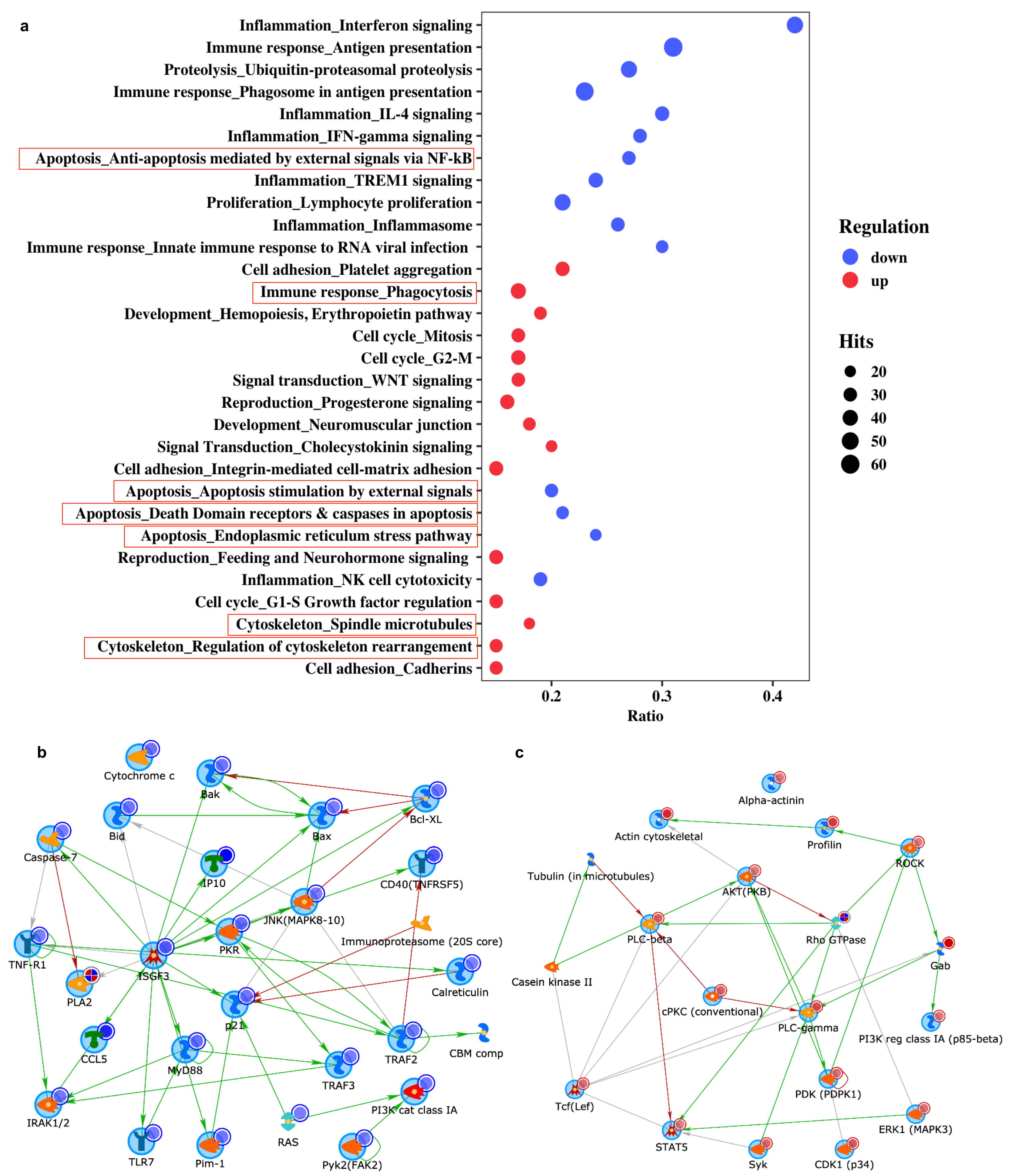

Supplementary Figure 8. Unbiased RNAseq analysis of *MAARS* knockdown in BMDMs. a Functional enrichment analysis of differentially expressed genes in BMDMs treated with MAARS or control gapmeRs (n=4 per group). Hits, represent the number of differentially expressed genes regulated per network. Color, denotes directionality of regulated genes in the network. Ratio, represents the number of regulated genes divided by the number of expected genes from each network. Shown are gene network visualizations of downregulated (**b**) and upregulated (**c**) pathways.
