# Supplementary material for: A macrophage-specific lncRNA regulates apoptosis and atherosclerosis by tethering HuR in the nucleus
Source: Nat Commun. 2020 Dec 1;11:6135. doi: 10.1038/s41467-020-19664-2 (PMC7708640; doi:10.1038/s41467-020-19664-2)
Supplement: Supplementary file 3 — Source Data [file 41467_2020_19664_MOESM3_ESM.pdf]

Fig. 1I

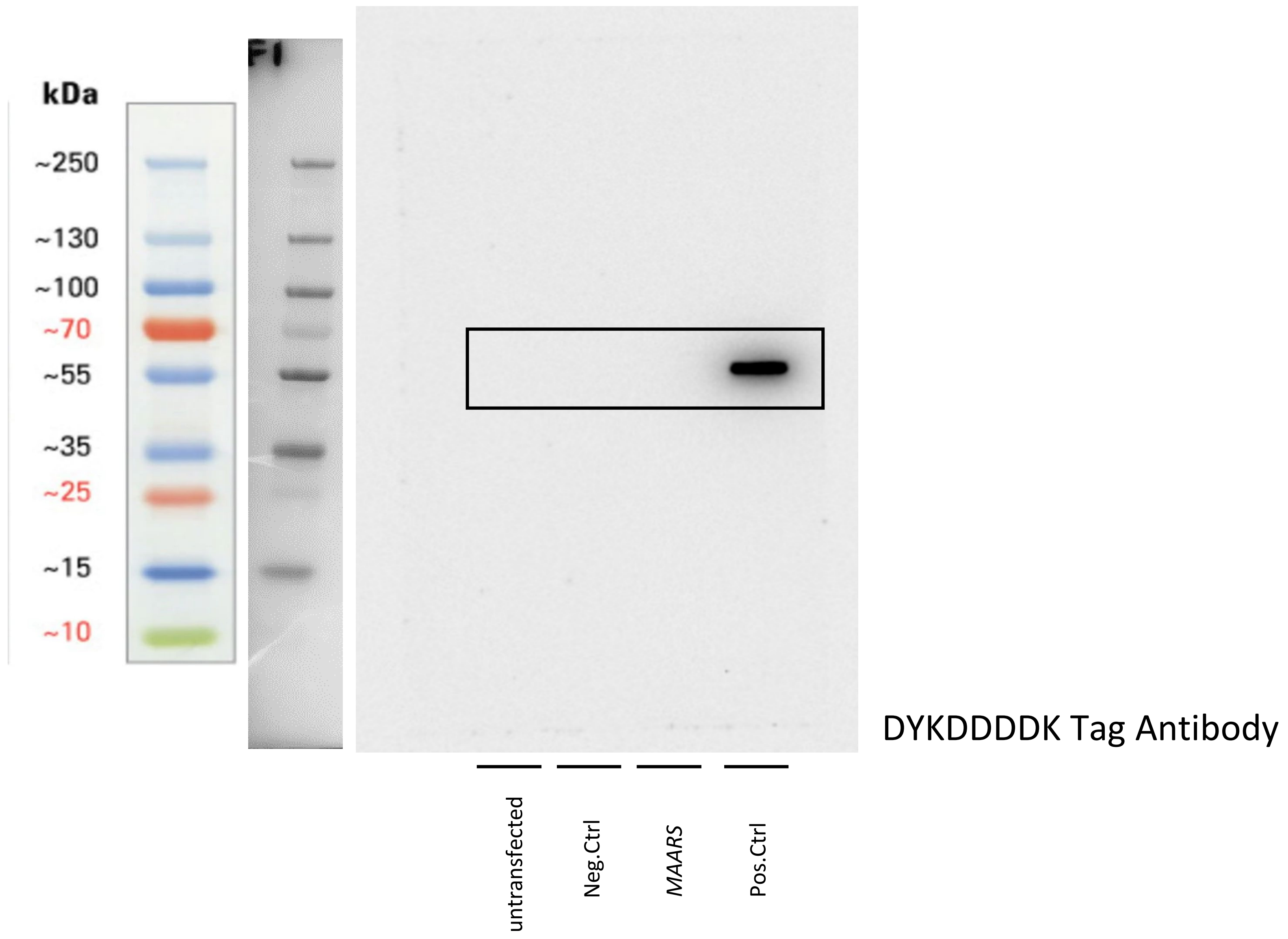
kDa
~250
~130
~100
~70
~55
~35
~25
~15
~10
DYKDDDDK Tag Antibody
untransfected
Neg.Ctrl
MAARS
Pos.Ctrl

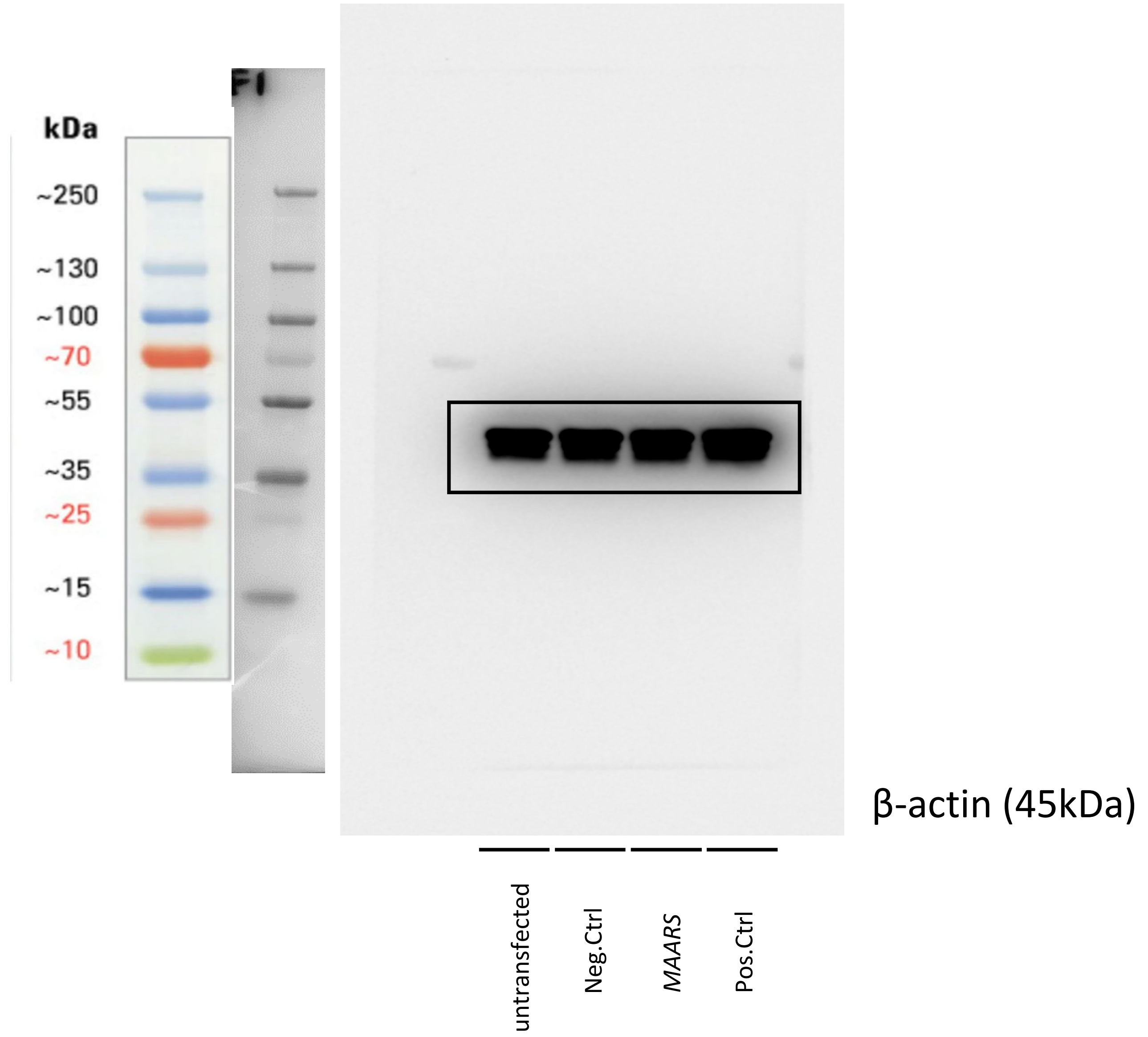
kDa
~250
~130
~100
~70
~55
~35
~25
~15
~10
β-actin (45kDa)
untransfected
Neg.Ctrl
MAARS
Pos.Ctrl

Fig. 3a

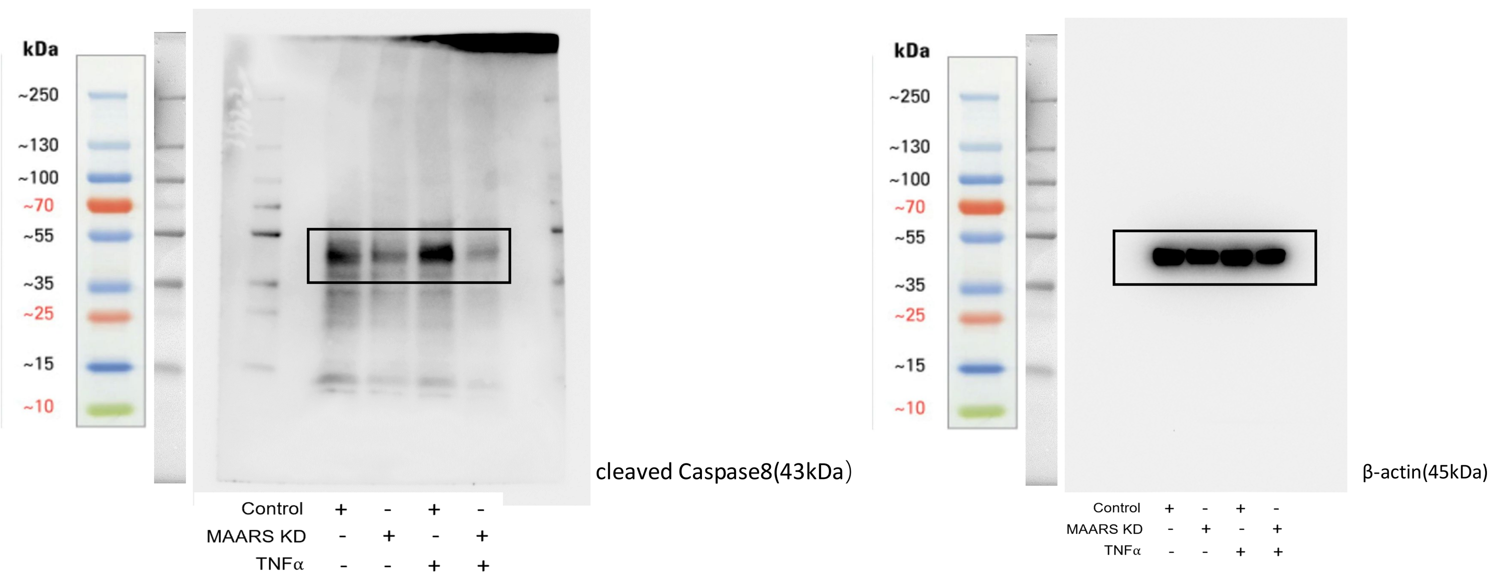

Fig. 3b

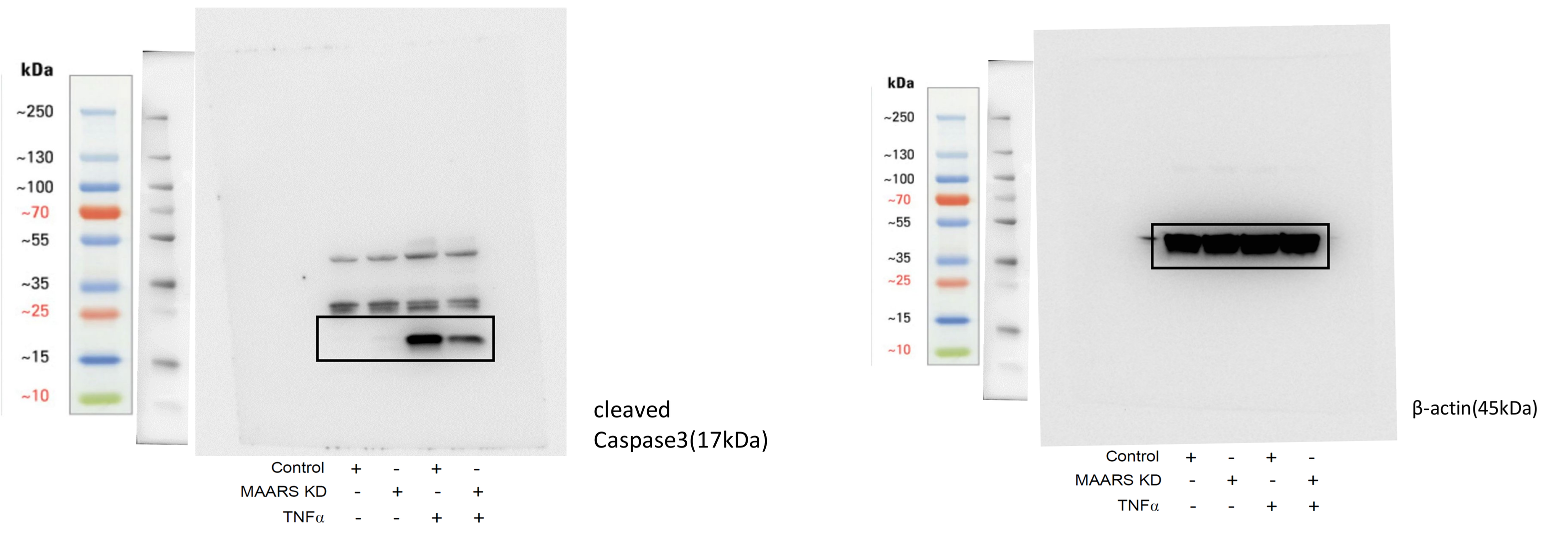

Fig. 3c

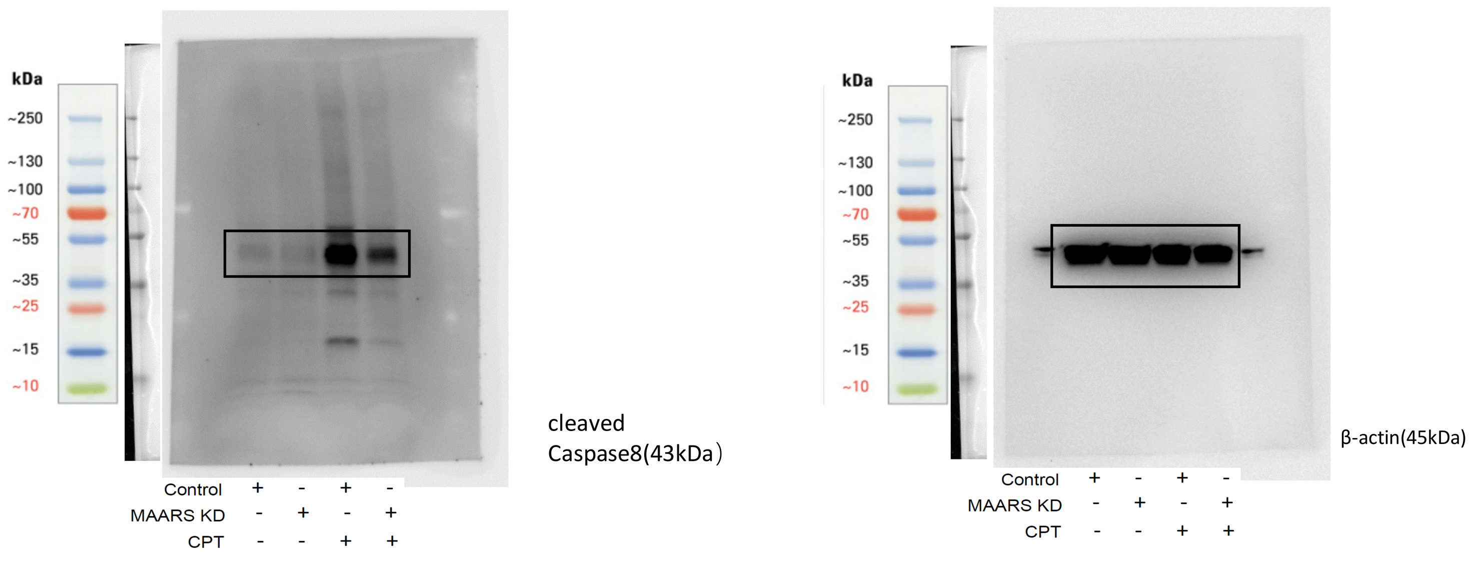

Fig. 3d

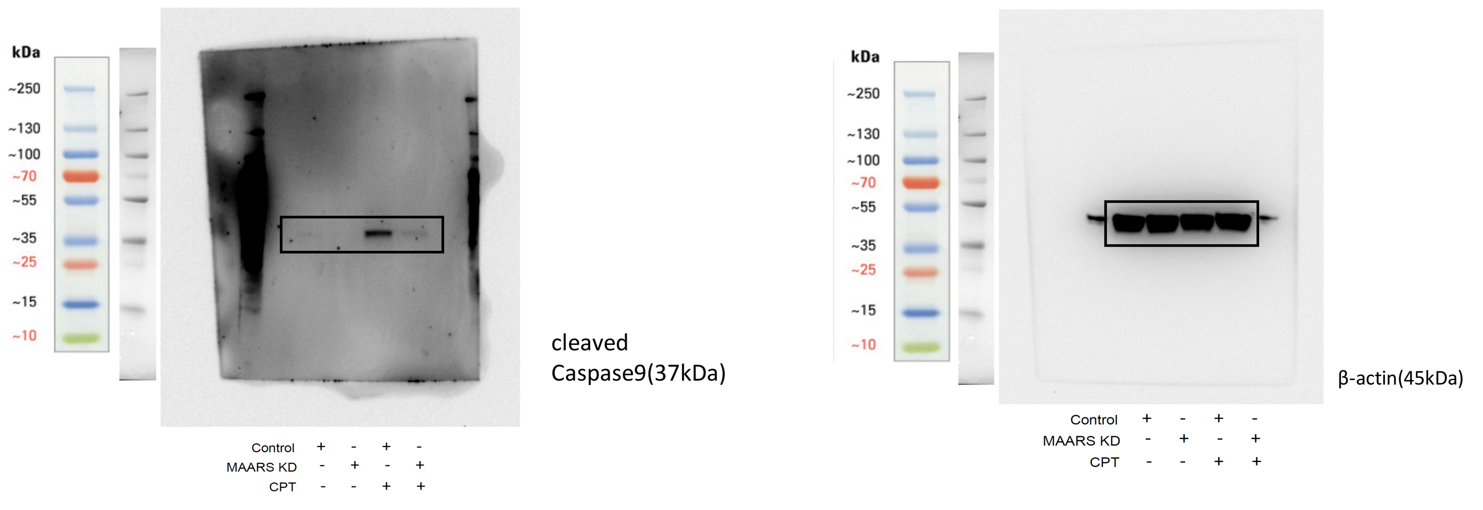

Fig. 3e

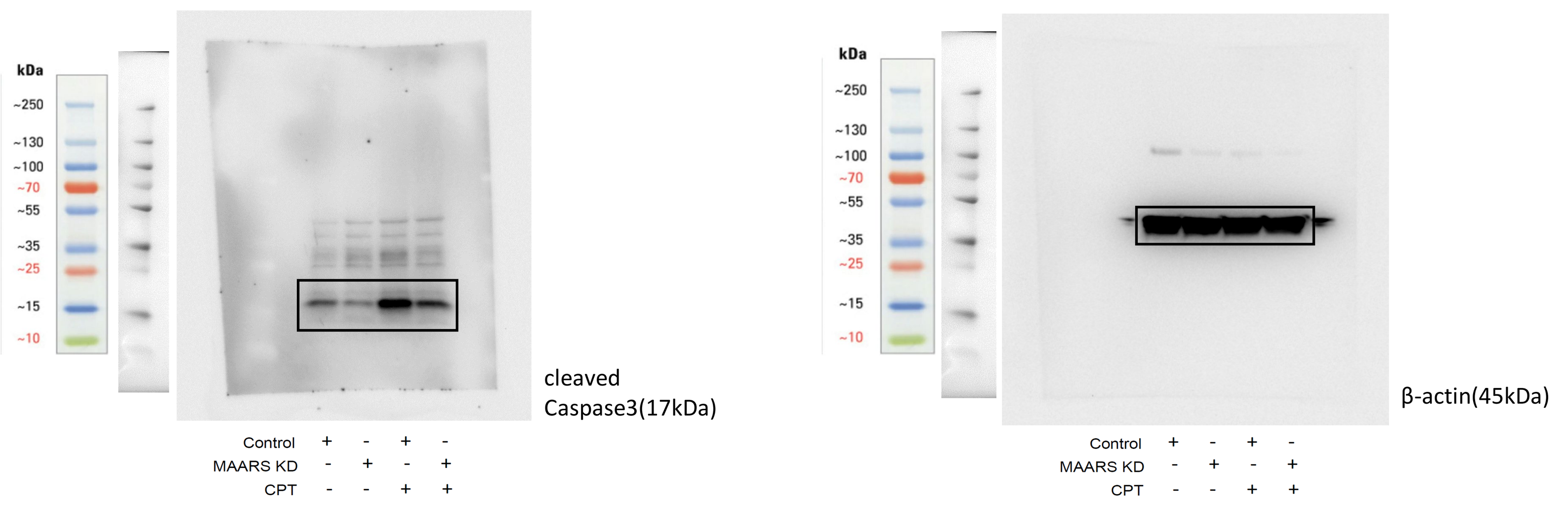

Fig. 3f-1

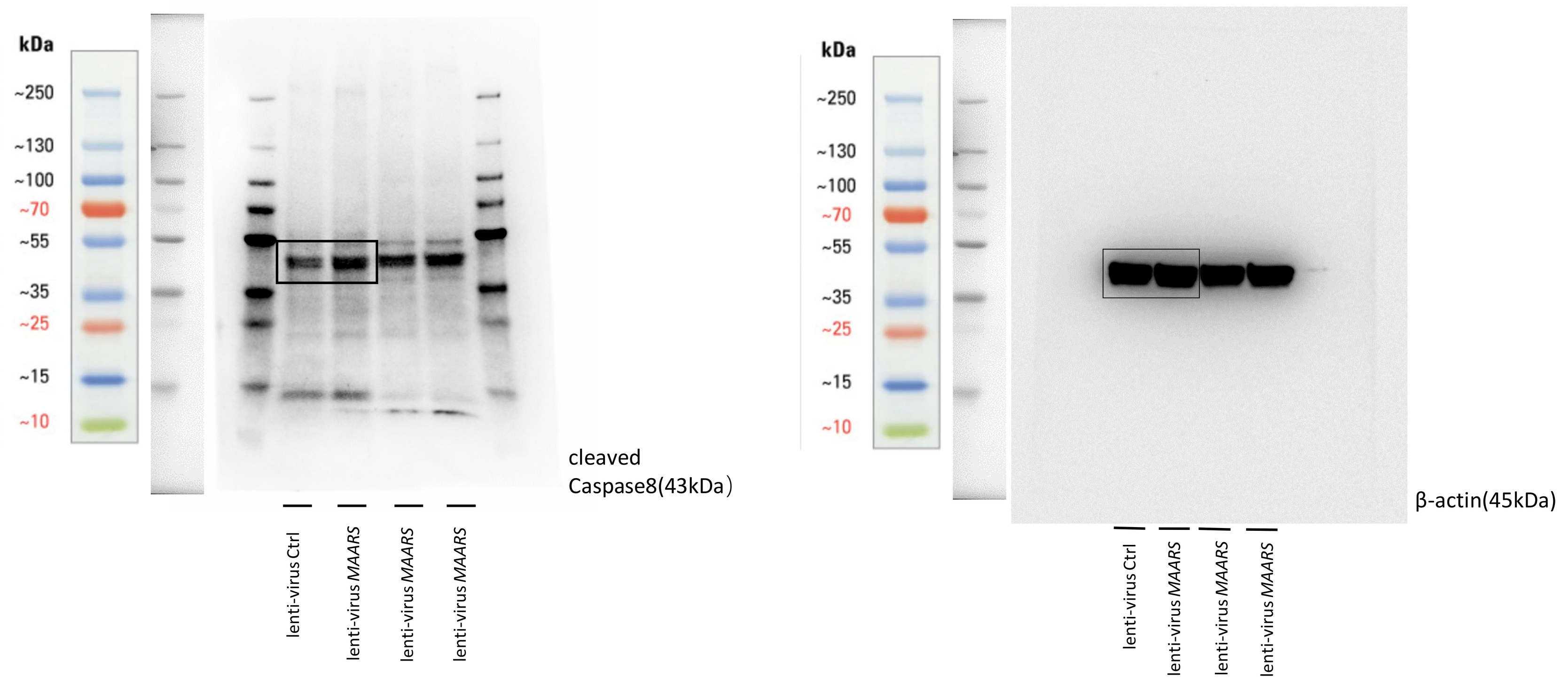

Fig. 3f-2

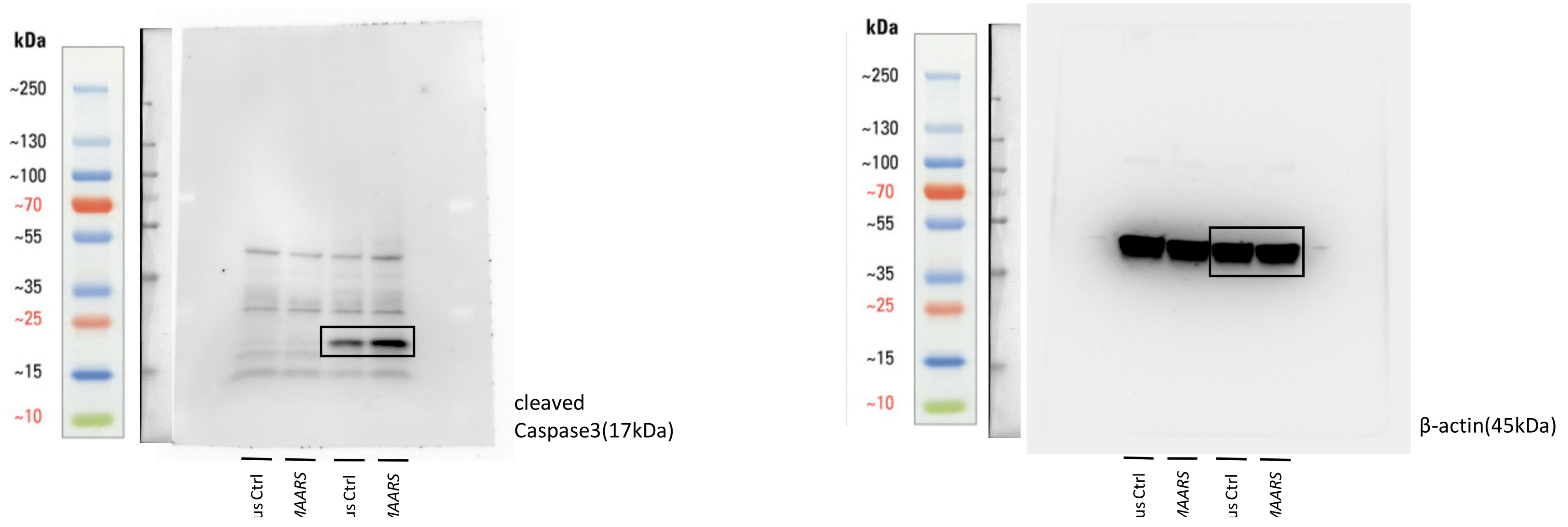

Fig. 4d

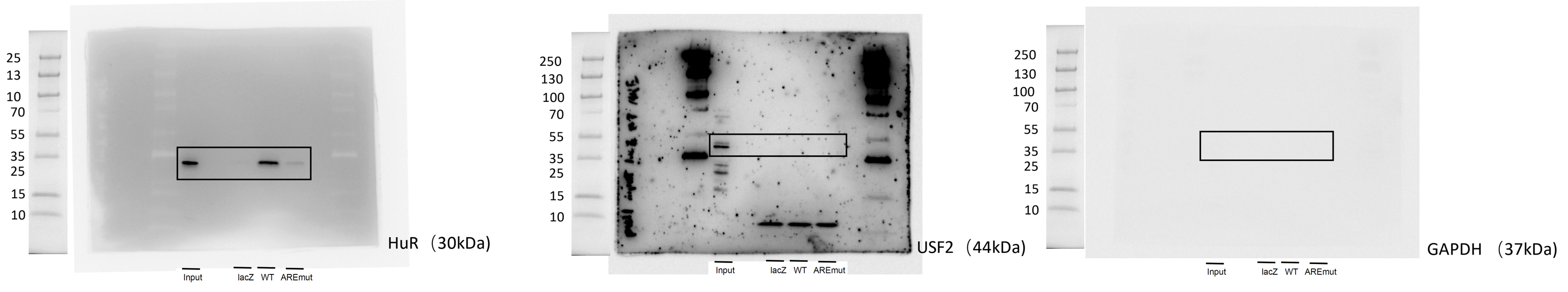

Fig. 4e

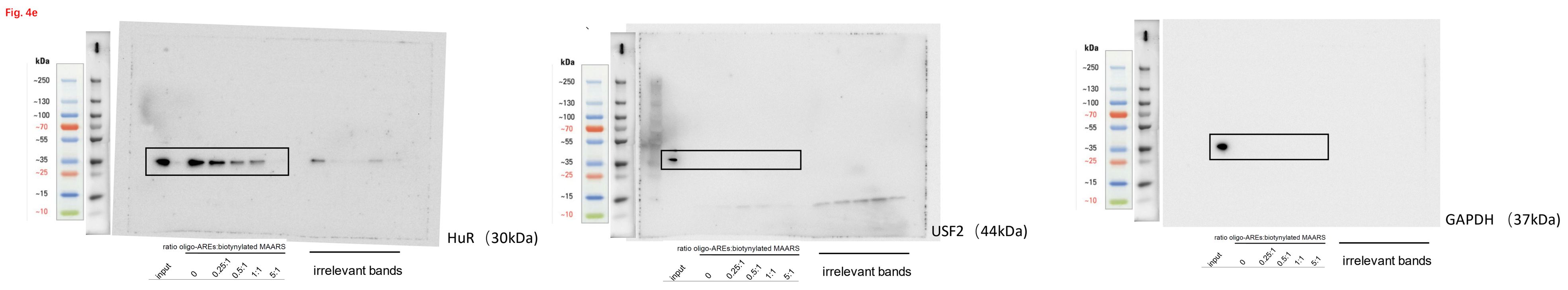

Fig. 4f

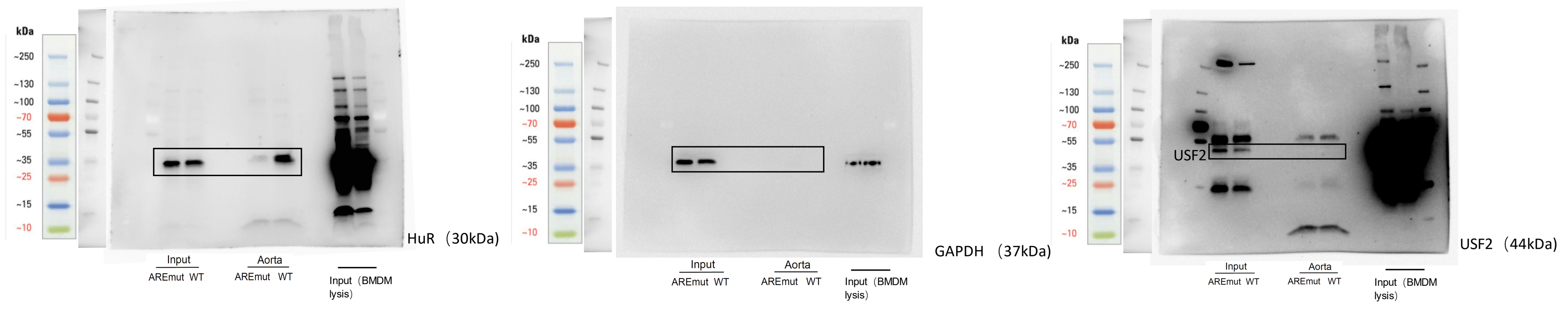

Fig. 4j

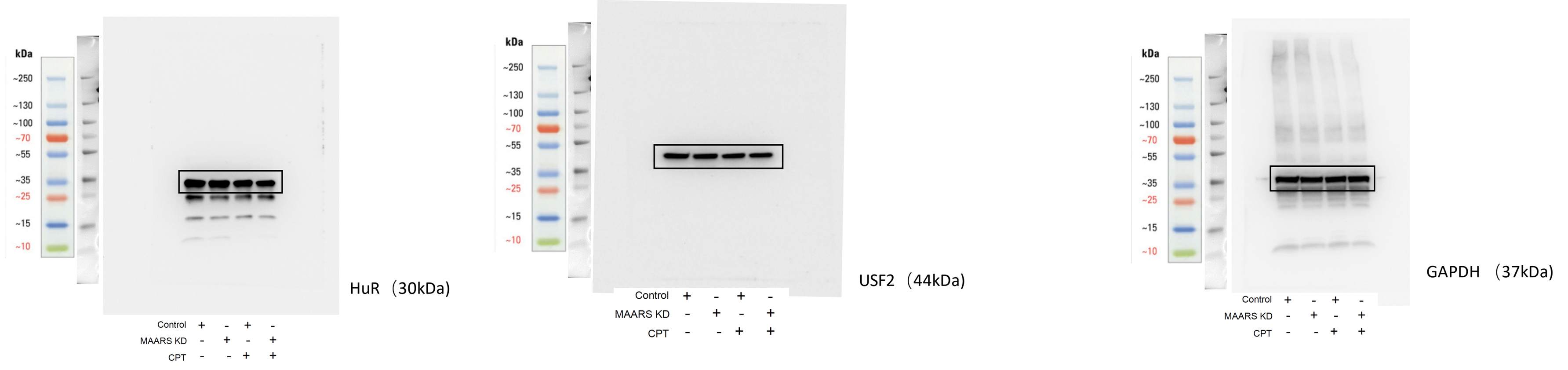

Fig. 4k

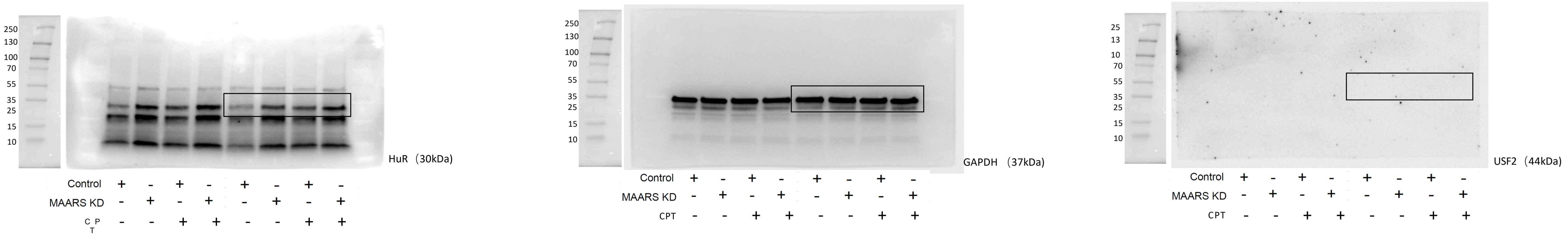

l

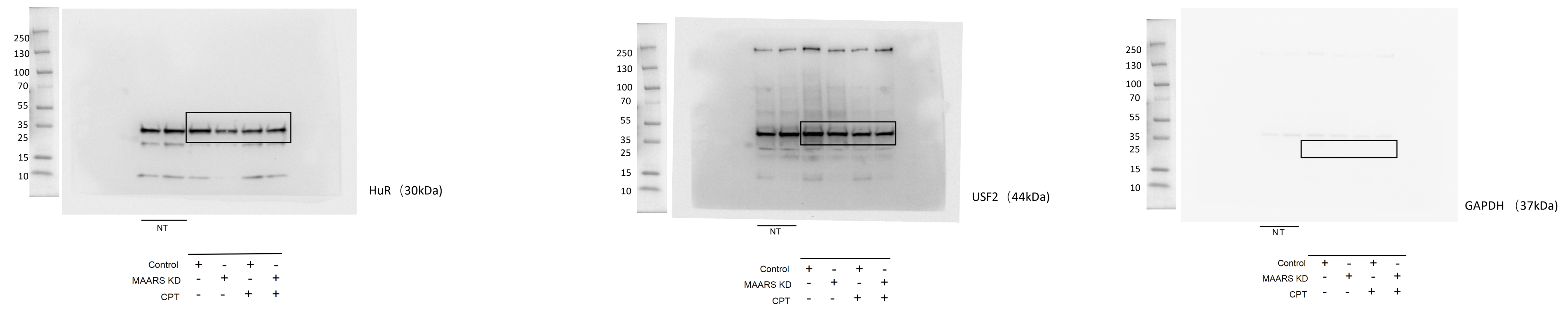

Fig. 5f

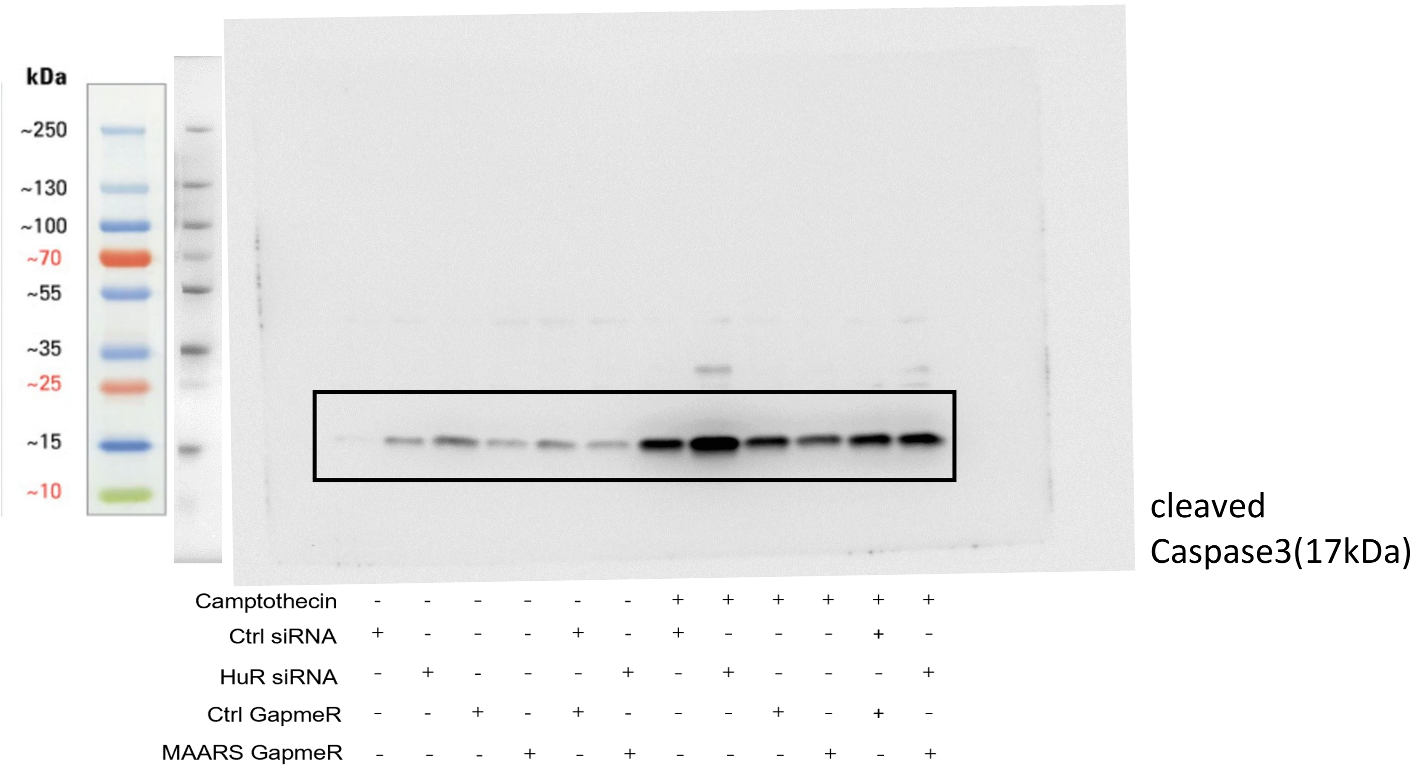
kDa
~250
~130
~100
~70
~55
~35
~25
~15
~10
cleaved Caspase3(17kDa)
Camptothecin - - - - - - + + + + + +
Ctrl siRNA + - - - + - + - - - + -
HuR siRNA - + - - - + - + - - - +
Ctrl GapmeR - - + - + - - - + - + -
MAARS GapmeR - - - + - + - - - + - +

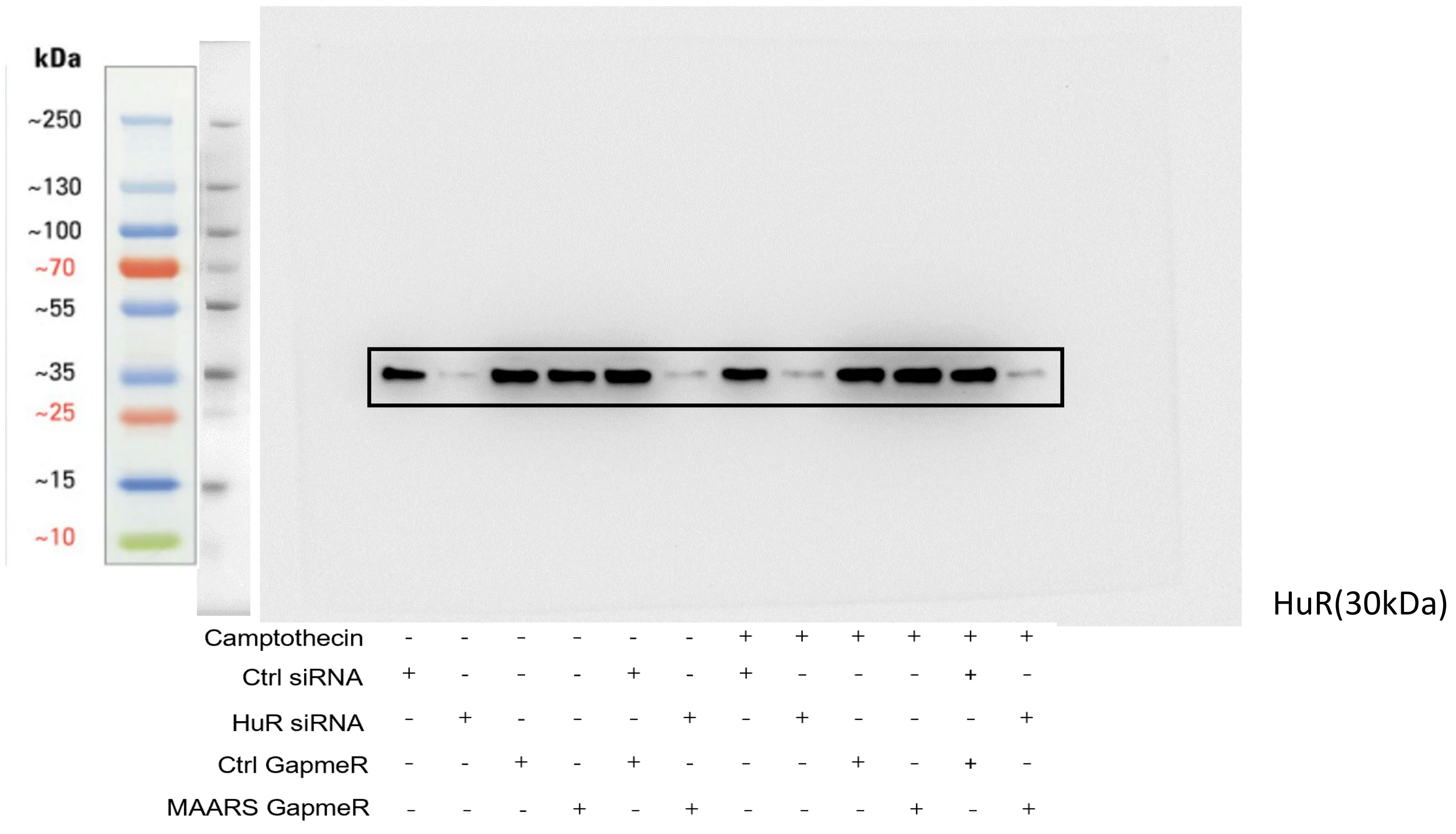
kDa
~250
~130
~100
~70
~55
~35
~25
~15
~10
HuR(30kDa)
Camptothecin - - - - - - + + + + + +
Ctrl siRNA + - - - + - + - - - + -
HuR siRNA - + - - - + - + - - - +
Ctrl GapmeR - - + - + - - - + - + -
MAARS GapmeR - - - + - + - - - + - +

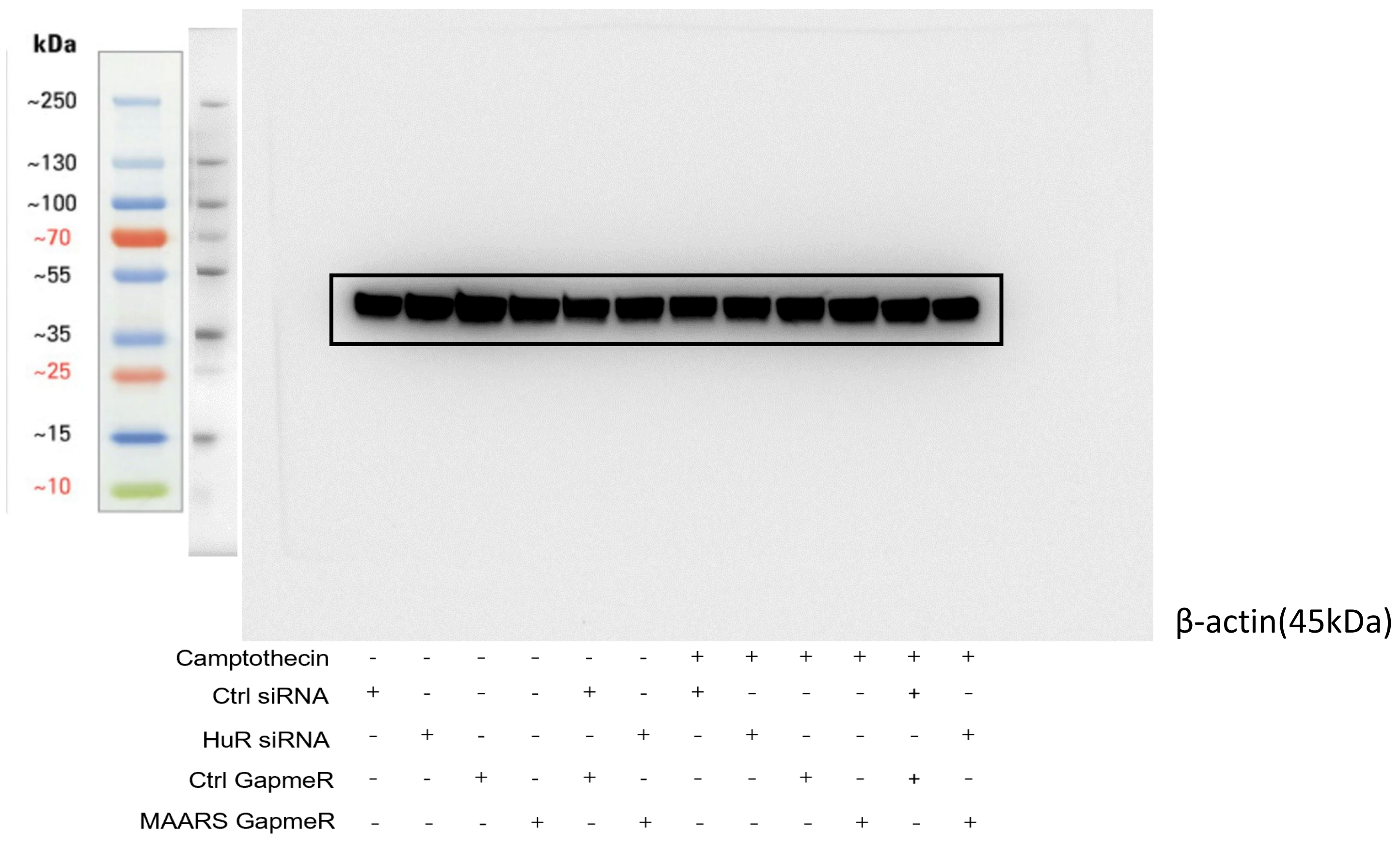
kDa
~250
~130
~100
~70
~55
~35
~25
~15
~10
β-actin(45kDa)
Camptothecin - - - - - - + + + + + +
Ctrl siRNA + - - - + - + - - - + -
HuR siRNA - + - - - + - + - - - +
Ctrl GapmeR - - + - + - - - + - + -
MAARS GapmeR - - - + - + - - - + - +

Suppl. 4i

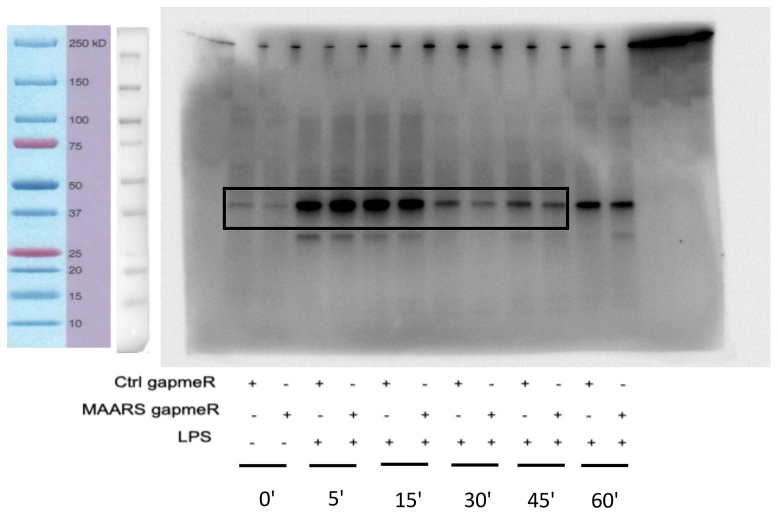

p-IκBα
（40kDa）

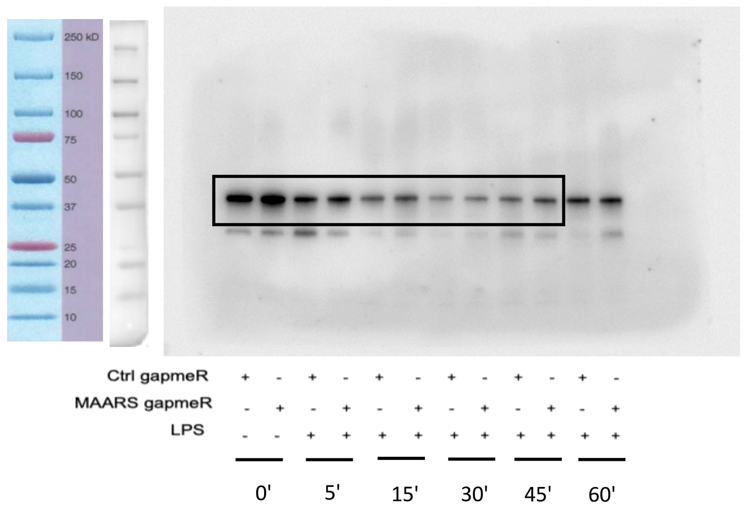

IκBα
（39kDa）

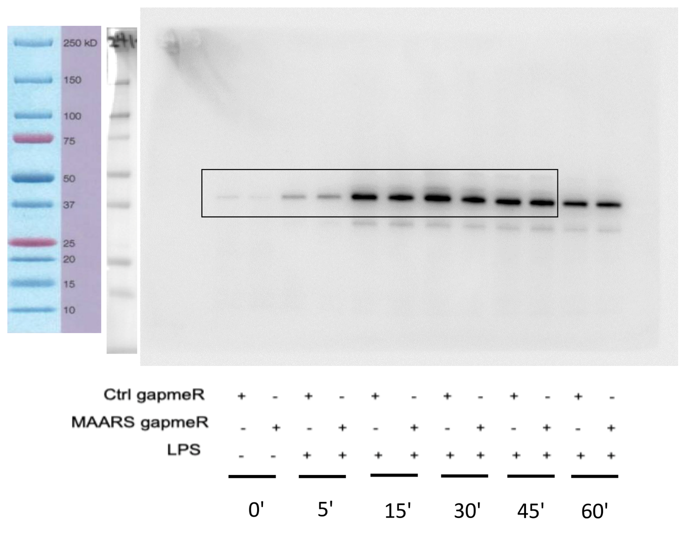

p-p38
（43kDa）

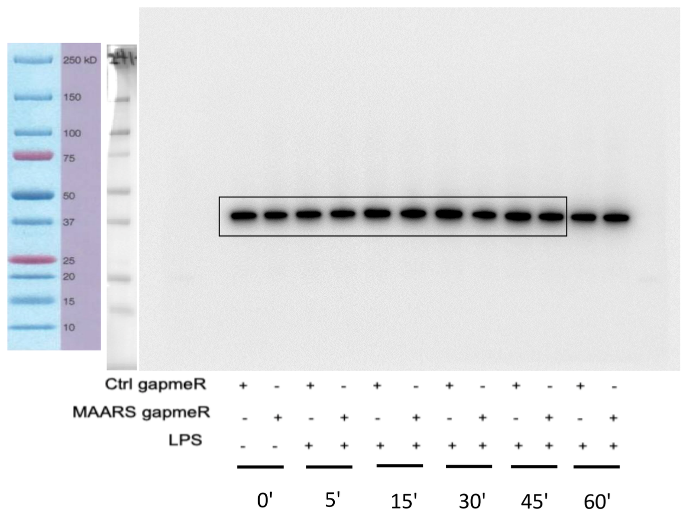

p38
（43kDa）

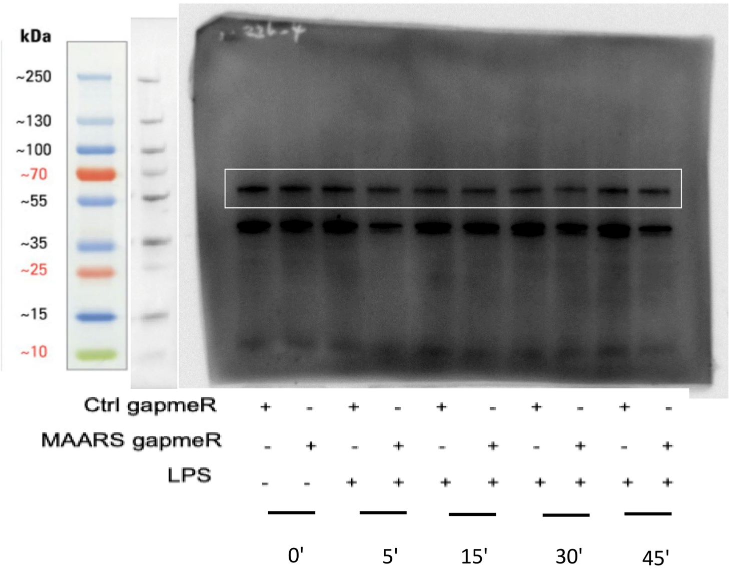

p-AKT
（60kDa）

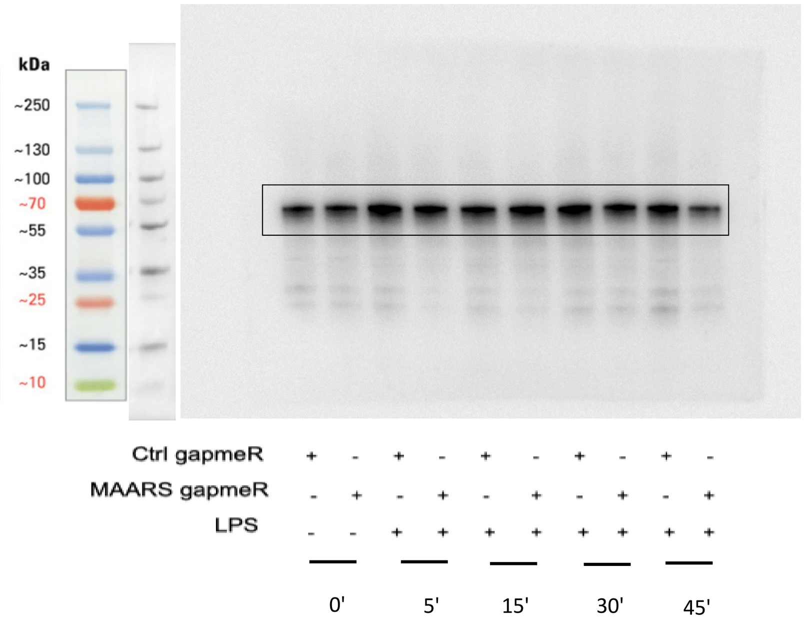

AKT
（60kDa）

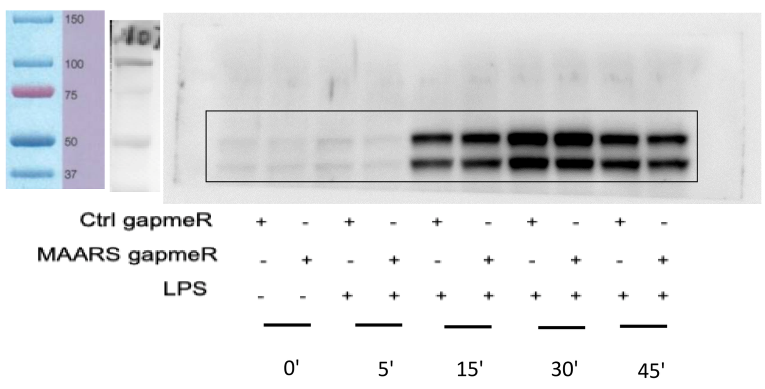

p-JNK(46, 54kDa)

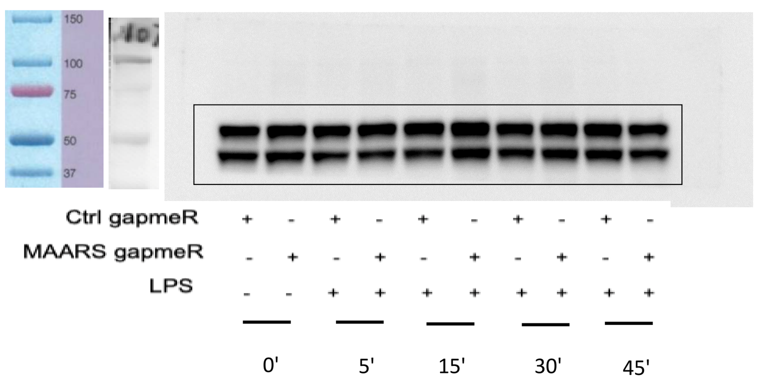

JNK(46, 54kDa)

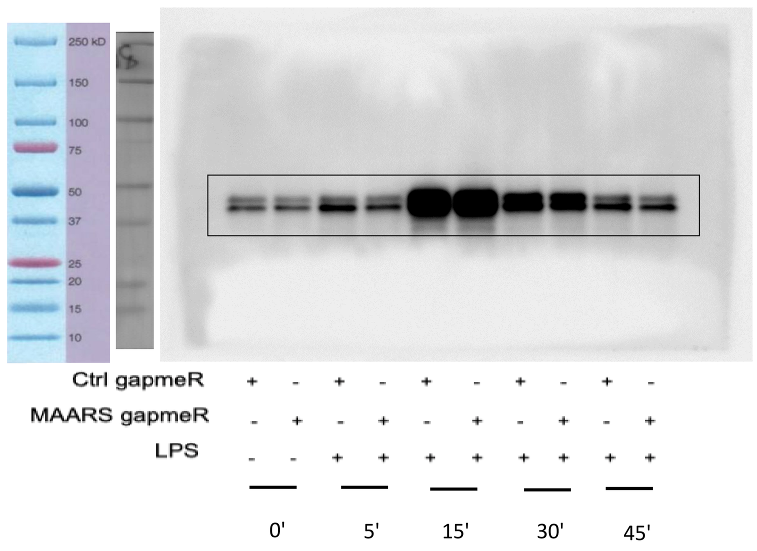

p-ERK1/2(44, 42kDa)

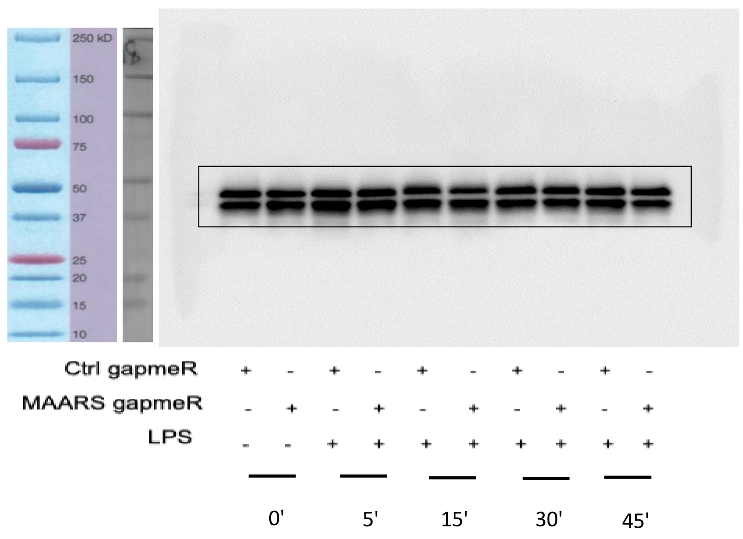

ERK1/2(44, 42kDa)

Suppl. 5b

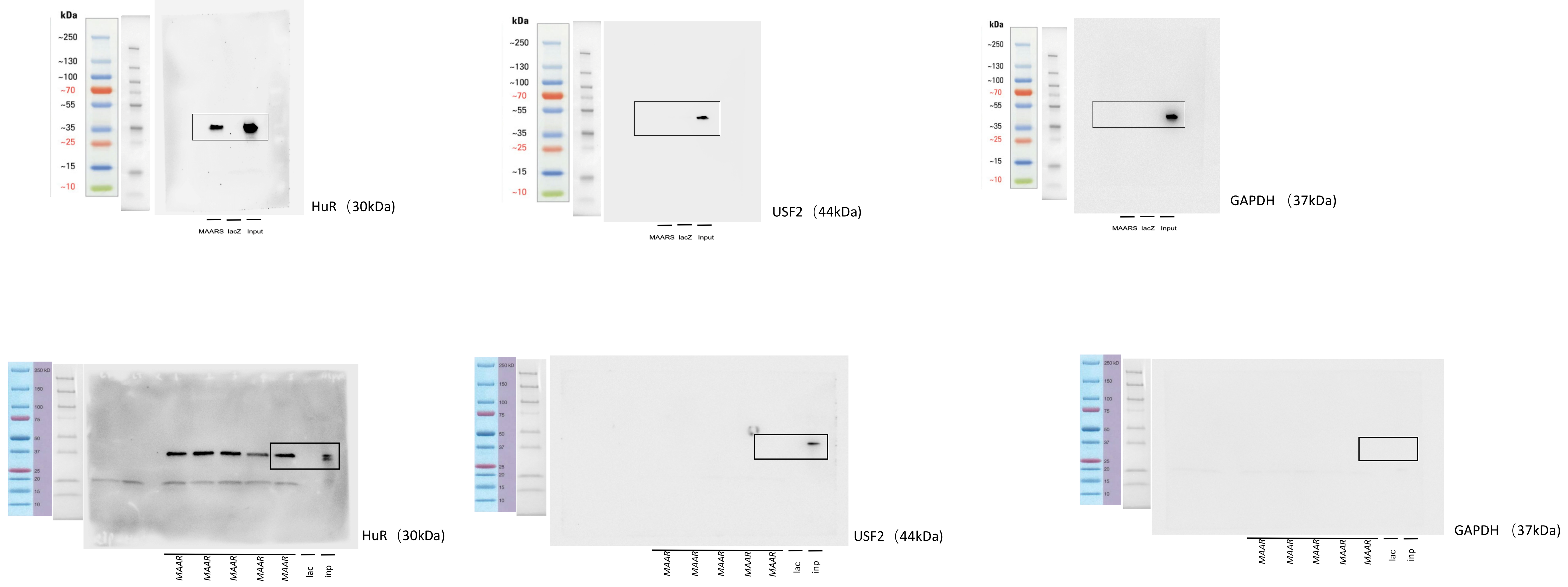

Suppl. 5c

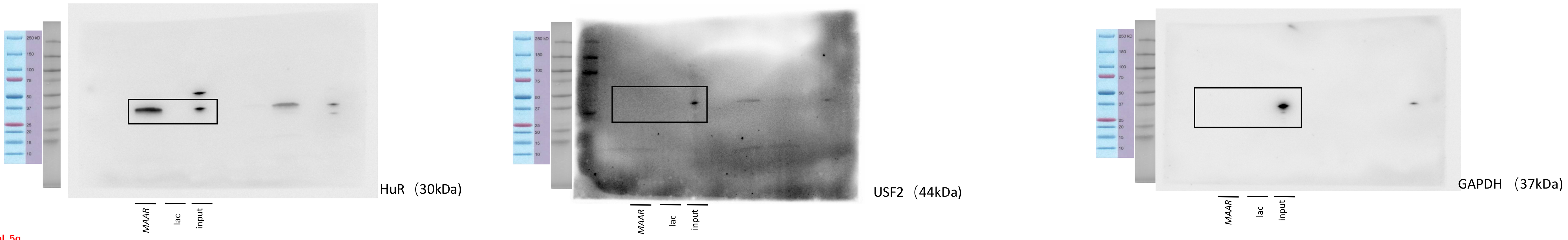

Suppl. 5g

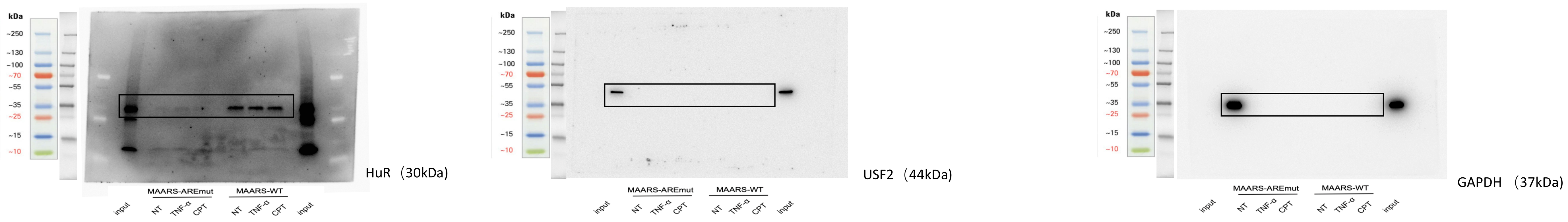

Suppl. 5h

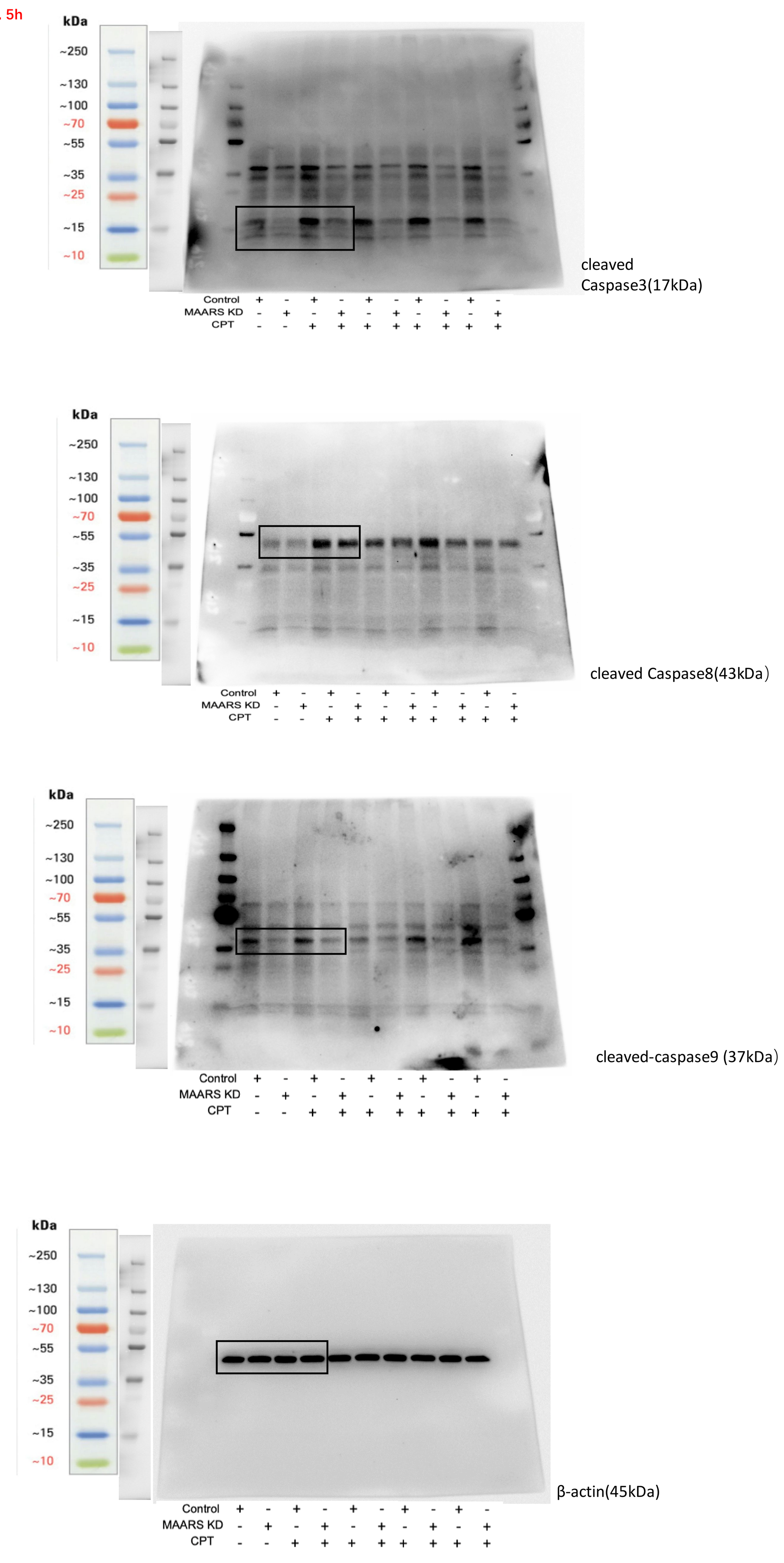

Suppl. 6f

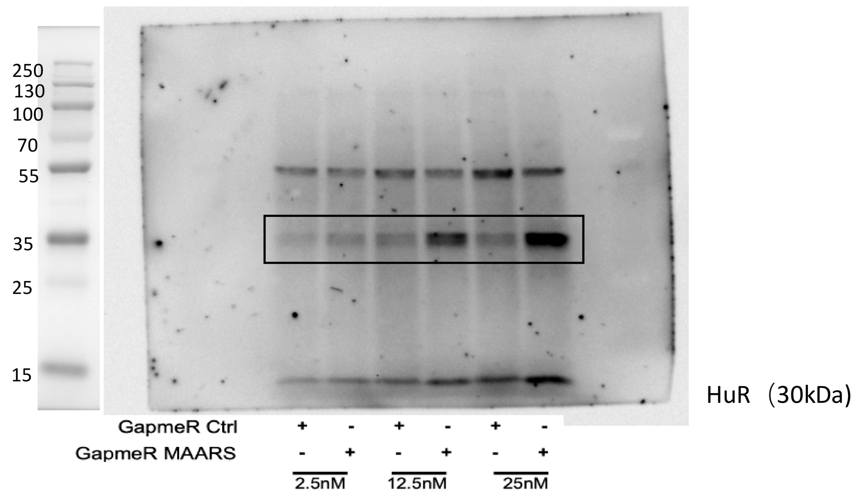
250
130
100
70
55
35
25
15
HuR（30kDa)
GapmeR Ctrl + - + - + -
GapmeR MAARS - + - + - +
2.5nM
12.5nM
25nM

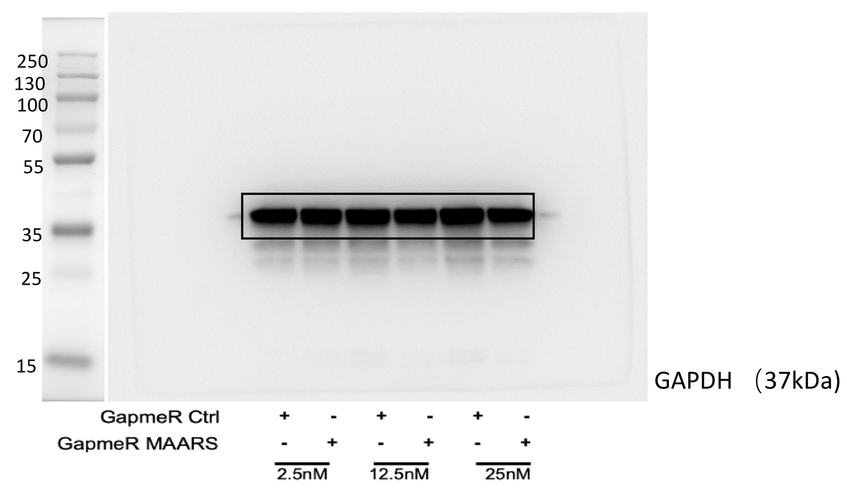
250
130
100
70
55
35
25
15
GAPDH （37kDa)
GapmeR Ctrl + - + - + -
GapmeR MAARS - + - + - +
2.5nM
12.5nM
25nM

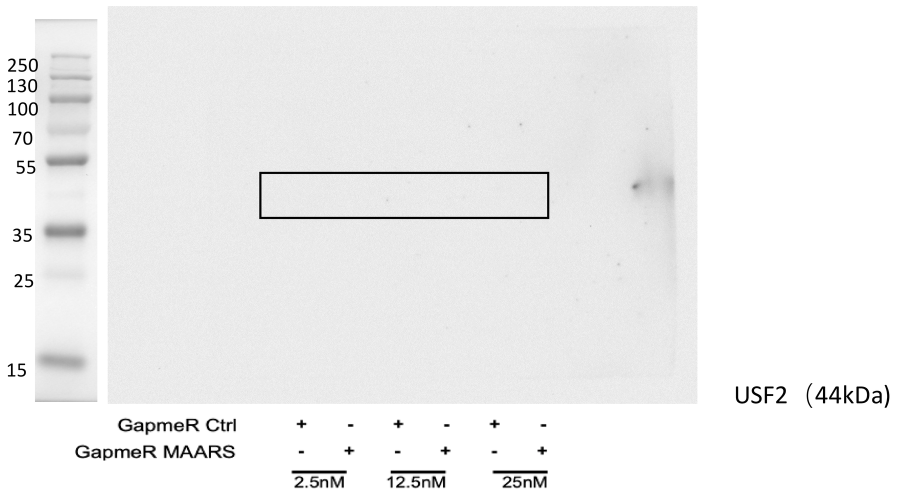
250
130
100
70
55
35
25
15
USF2（44kDa)
GapmeR Ctrl + - + - + -
GapmeR MAARS - + - + - +
2.5nM
12.5nM
25nM

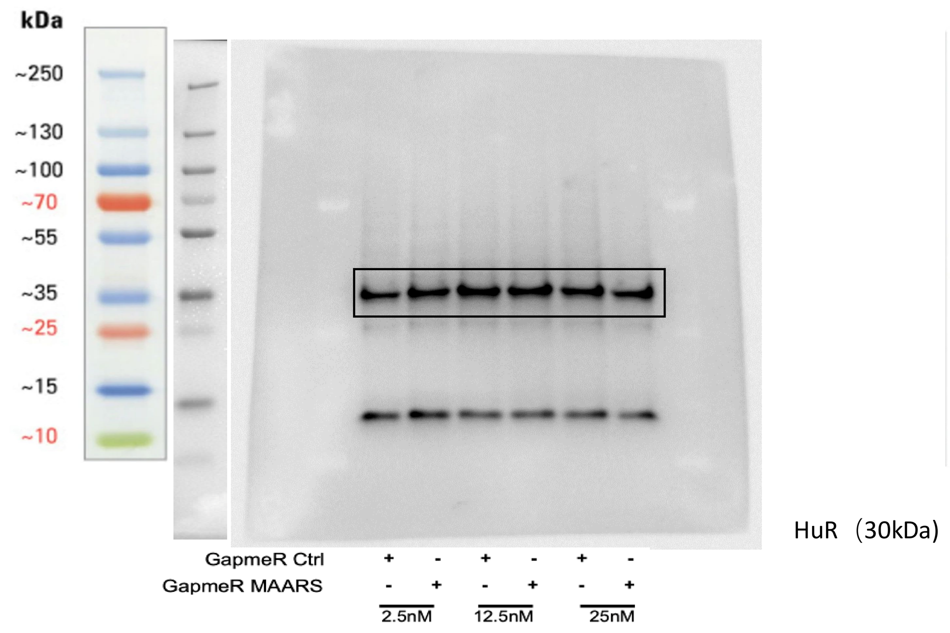
kDa
~250
~130
~100
~70
~55
~35
~25
~15
~10
HuR（30kDa)
GapmeR Ctrl + - + - + -
GapmeR MAARS - + - + - +
2.5nM
12.5nM
25nM

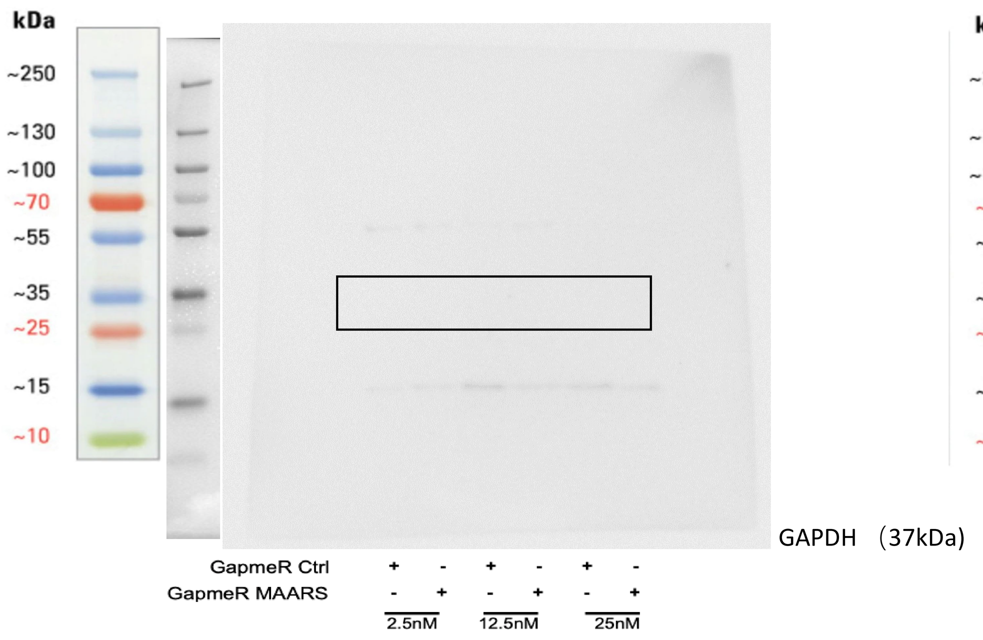
kDa
~250
~130
~100
~70
~55
~35
~25
~15
~10
GAPDH （37kDa)
GapmeR Ctrl + - + - + -
GapmeR MAARS - + - + - +
2.5nM
12.5nM
25nM

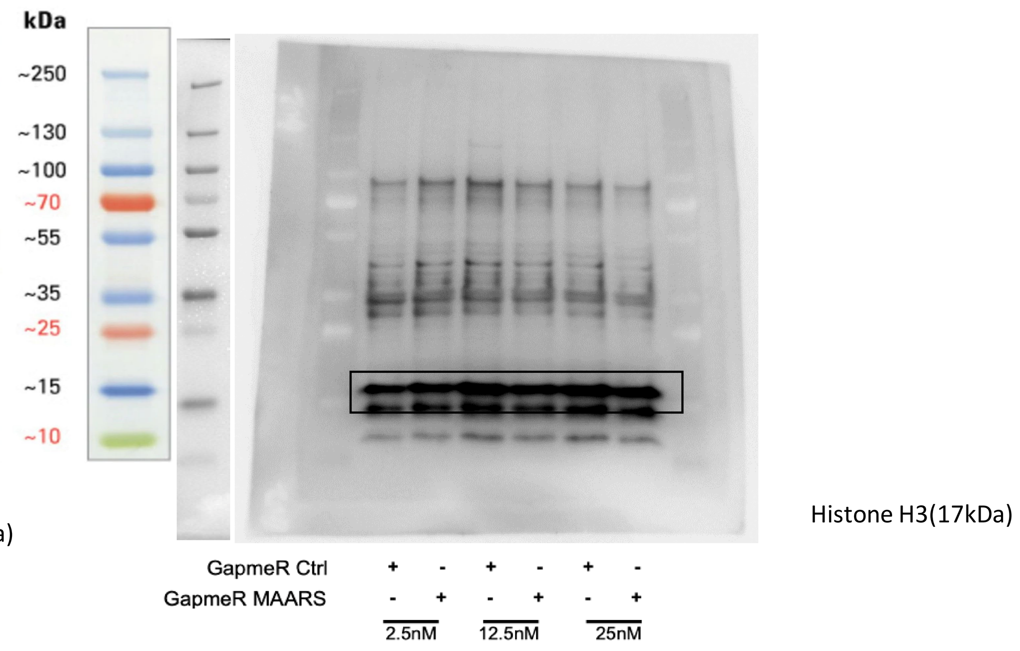
kDa
~250
~130
~100
~70
~55
~35
~25
~15
~10
Histone H3(17kDa)
GapmeR Ctrl + - + - + -
GapmeR MAARS - + - + - +
2.5nM
12.5nM
25nM

Suppl. 7e

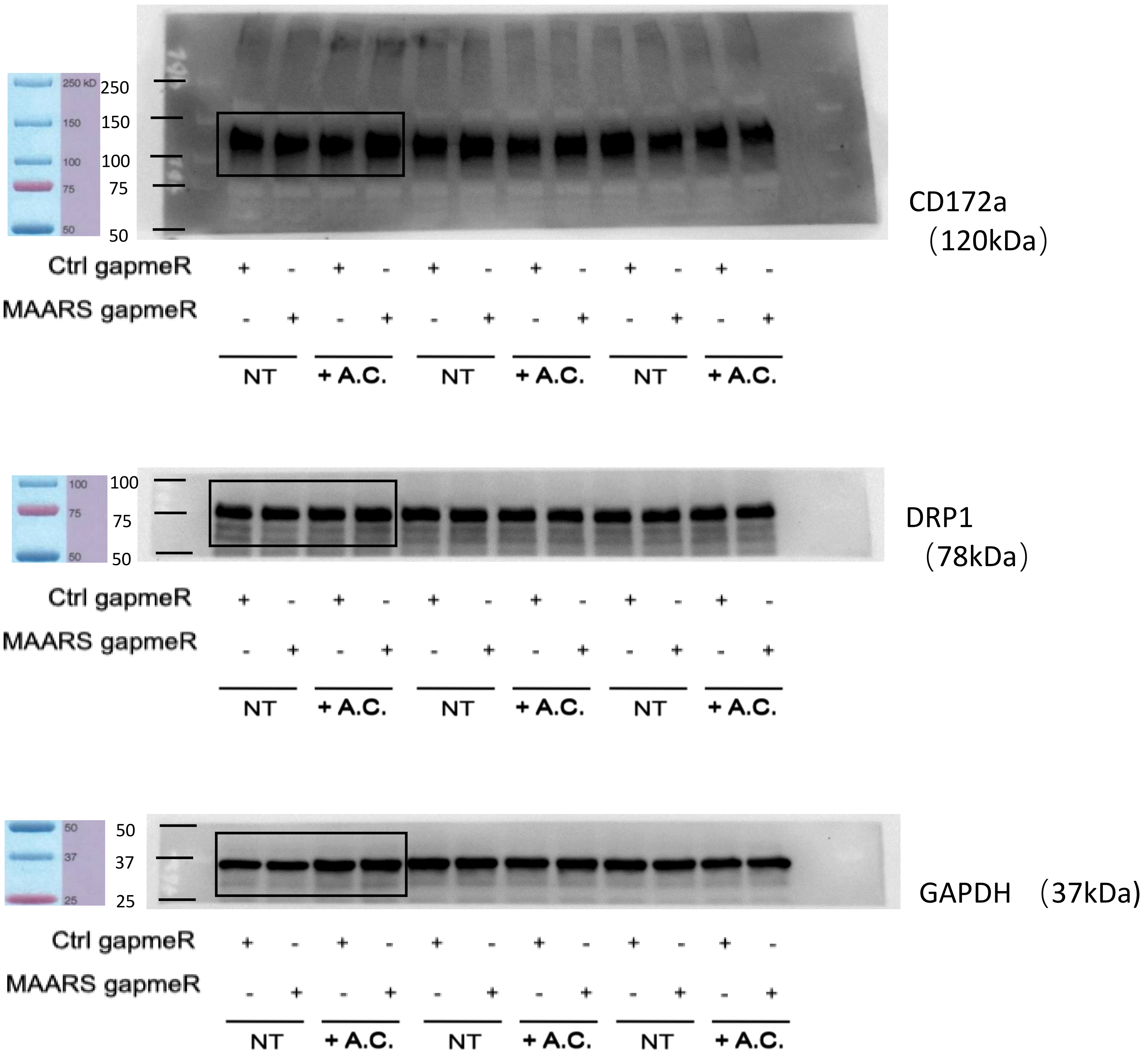
250
150
100
75
50
CD172a
(120kDa)
Ctrl gapmeR + - + - + - + - + - + -
MAARS gapmeR - + - + - + - + - + - +
NT
+ A.C.
NT
+ A.C.
NT
+ A.C.
100
75
50
DRP1
(78kDa)
Ctrl gapmeR + - + - + - + - + - + -
MAARS gapmeR - + - + - + - + - + - +
NT
+ A.C.
NT
+ A.C.
NT
+ A.C.
50
37
25
GAPDH (37kDa)
Ctrl gapmeR + - + - + - + - + - + -
MAARS gapmeR - + - + - + - + - + - +
NT
+ A.C.
NT
+ A.C.
NT
+ A.C.
